# Presenting Antimicrobial Peptides on Poly(ethylene glycol): Star-Shaped vs Comb-Like Architectures

**DOI:** 10.1021/acs.macromol.4c02762

**Published:** 2025-02-05

**Authors:** Zixian Cui, Elliot A. Brna, Matthew A. Crawford, Puthayalai Treerat, Mobina Alimadad, Molly A. Hughes, Rachel A. Letteri

**Affiliations:** † Department of Chemical Engineering, University of Virginia, Charlottesville, Virginia 22903, United States; ‡ Division of Infectious Diseases & International Health, Department of Medicine, University of Virginia, Charlottesville, Virginia 22908, United States

## Abstract

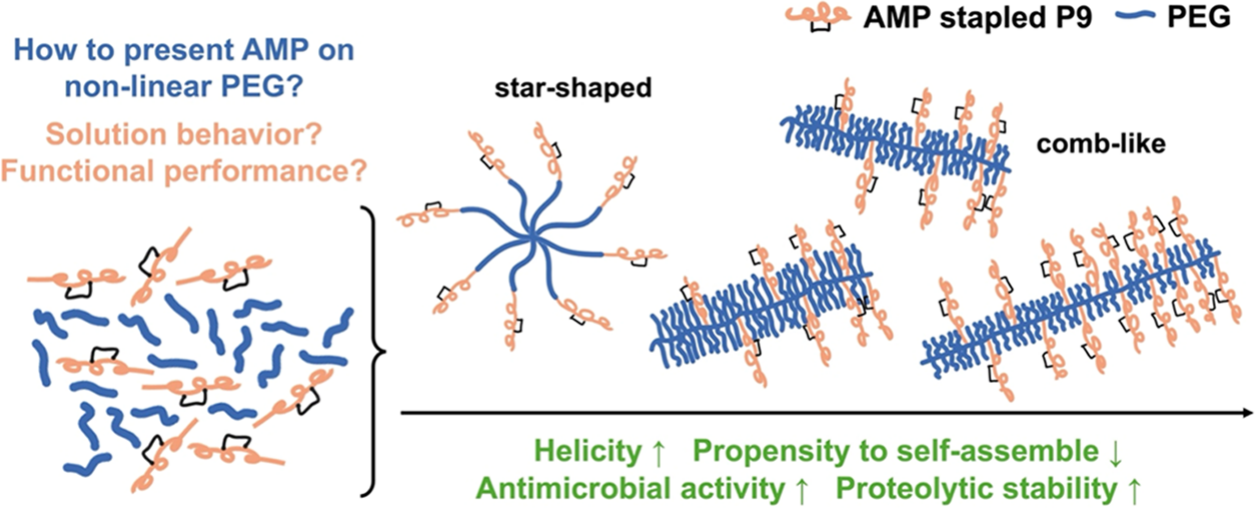

Conjugating antimicrobial peptides (AMPs) to nonlinear polymers is a promising strategy to overcome the translational challenges of AMPs toward treating infections caused by antibiotic-resistant bacteria. Nonlinear polymers, and therefore conjugates, can be prepared with various architectures (e.g., star-shaped, comb-like, hyperbranched, etc.), however, the effects of polymer architecture on antimicrobial performance and related properties, like size and morphology in solution and secondary structure, are not yet well-understood. Here, we compare conjugates of the human chemokine-derived AMP stapled P9 with poly(ethylene glycol) (PEG) prepared in two of the major nonlinear architectures: star-shaped and comb-like. At comparable molecular weights and compositions (peptide wt %), comb-like conjugates afford increased helicity, solubility, antimicrobial activity, and proteolytic stability compared to star-shaped analogs. We then leveraged the expansive design space of comb-like architectures to prepare conjugates with different backbone lengths and PEG side chain lengths, with shorter PEG side chains leading to increased helicity, yet potentially less shielding from proteolytic degradation and the longest backbone lengths furnishing the most potent antimicrobial activity. Both comb-like and star-shaped conjugates display high zeta potential, indicating that the cationic AMPs were accessible for electrostatic interactions with bacterial membranes. Yet, the comb-like conjugates showed a higher fraction of unimolecular structures indicative of a lower propensity for supramolecular assembly that could be encumbering the desired AMP-bacteria interactions in the star-shaped conjugates. Together, our work shows comb-like AMP-polymer conjugates to outperform analogous star-shaped conjugates, while adding design flexibility to access an expansive range of monomer chemistries, monomer distributions, and backbone lengths to modulate performance-determining properties and ultimately furnish an effective suite of AMP-polymer materials as alternatives to conventional antibiotics for combatting bacterial infections.

## Introduction

Antimicrobial peptides (AMPs) are emergent promising alternatives to conventional antibiotics for treating infections caused by antibiotic-resistant bacteria.^1,2^ Combining hydrophobic and cationic character, many AMPs can interact selectively with the anionic cell envelopes of bacteria over the more neutral membranes of mammalian cells.^3,4^ However, the clinical implementation of AMPs is limited by their solubility, rapid clearance, proteolytic degradation, and toxicity.^5^ Presenting AMPs on polymers provides unique opportunities to overcome these hurdles and enhance the functional performance of AMPs by tuning composition, molecular weight, and molecular architecture.^6−11^

Typically, the polymer components of AMP-polymer conjugates are cationic and/or charge-neutral to strike a balance between the cationic, hydrophobic character needed for bacterial killing and the neutral, hydrophilic character that confers biocompatibility.^8,9,12^ Cationic polymers like poly(ethylenimine) (PEI)^13^ and poly(amido amine) (PAMAM)^14^ increase the solubility and potency of AMPs, yet often add toxicity through multivalent electrostatic interactions with slightly anionic mammalian cell membranes. On the other hand, charge-neutral hydrophilic polymers, like poly(ethylene glycol) (PEG),^15^ are less toxic to mammalian cells, yet can reduce or even abrogate AMP-mediated antimicrobial activity by shielding desired AMP interactions with bacterial membranes.^16,17^ Encouragingly, increasing the density of AMPs presented on neutral hydrophilic polymers, such as when AMP-PEG conjugates supramolecularly assemble into micelles or nanoparticles with peptide cores and PEG shells, enhances AMP compatibility with mammalian cells while maintaining antimicrobial activity.^18,19^ Thus, to fully integrate the benefits of both the antimicrobial activity from the AMP and the biocompatibility from the charge-neutral polymer, it is important to understand how AMP-polymer conjugate design features impact assembly in solution and ultimately antimicrobial performance.

In addition to composition, polymer architecture is a critical determiner of solution properties and functional performance.^6,20−23^ AMP-polymer conjugates with nonlinear architectures (e.g., star-shaped and comb-like) accommodate multiple AMPs on a single molecule and provide opportunities to present AMPs at high density to better balance antimicrobial activity and biocompatibility as compared to their linear analogs and/or unconjugated AMPs. For example, increasing the number of AMPs on star-shaped^13,24^ or comb-like conjugates^25,26^ increases antimicrobial activity–in some cases^13,25^ without accompanying increases in toxicity. Since most studies focus on comparing different design features within one principal architecture, comparisons across different architectures, but with similar compositions and molecular weights, are needed to guide the design of AMP-polymer conjugates.

A notable benefit of comb architectures is the design flexibility, allowing the variation of monomer composition, side chain length, AMP density, backbone length, and other factors. Therefore, in addition to comparing star-shaped to comb-like architectures, we examine the effects of PEG side chain length on comb-like conjugates, motivated by our prior work showing that shortening the PEG arms in star-shaped conjugates enhances antimicrobial activity.^27^ Given that shorter PEG side chains are more hydrophobic,^28^ they could enhance interactions between the conjugates and bacteria, as well as shield AMPs from proteases. Alternatively, the lower steric bulk of the shorter side chains may leave AMPs more accessible, which may benefit performance by increasing antimicrobial activity or decrease the performance by permitting access to degrading proteases. We also vary polymer backbone length at constant peptide density, anticipating that concentrating more AMPs onto a single chain will increase antimicrobial activity.

In this work, we use reversible addition–fragmentation chain transfer (RAFT) polymerization of PEG- and AMP-functionalized monomers to prepare comb-like conjugates with similar molecular weights and compositions to two of the star-shaped AMP-PEG conjugates we reported previously.^27^ We then leverage the additional design flexibility associated with comb polymers to vary the backbone length and PEG side chain length. Holding the concentration of AMP constant, we characterized the solution properties (i.e., secondary structure, size, zeta potential, and morphology) and antimicrobial performance (i.e., antimicrobial activity, hemolysis, and proteolytic resistance) of these investigational conjugates. Our study reveals changes in solution properties, stability, and AMP helicity with polymer architecture and PEG side chain length and highlights the merit of comb-like architectures, both from a performance and design flexibility perspective. By furthering our understanding of the connection between molecular features of AMP-polymer conjugates, their solution properties, and antimicrobial performance, we anticipate that our findings will contribute to accelerating the clinical development and implementation of AMPs by presenting them on polymers to combat antimicrobial-resistant microorganisms.

## Instrumentation and Methods

### Materials

Fmoc-l-Pro-OH (≥98%), Fmoc-l-Glu(OtBu)–OH (≥98%), Fmoc-l-Ser(Trt)–OH (≥98%), Fmoc-l-Arg(Pbf)–OH, Fmoc-l-Lys(Boc)–OH (≥98%), Fmoc-l-Leu-OH (≥98%), Fmoc-l-Val-OH (≥98%), Fmoc-(S)-2-(4-pentenyl)-Alanine–OH (≥97%), Fmoc-6-Ahx–OH, rink amide resin LS (0.5 mmol/g, 100–200 mesh), and oxyma pure (≥99%) were purchased from Advanced ChemTech. *N*,*N*-dimethylformamide (DMF, ≥99.8%), methacrylic anhydride (≥98%), piperidine (≥99%), diisopropylcarbodiimide (DIC, ≥99%), Grubbs Catalyst Generation I (M102, ≥97%), 1,2-dichloroethane (DCE) (≥99%), dichloromethane (DCM, ≥99.5%), triisopropylsilane (TIPS, ≥98%), diethyl ether (≥99%), 2,2′-(ethylenedioxy)diethanethiol (DODT, ≥95%), trifluoroacetic acid (TFA, ≥99%), acetonitrile (ACN, for high-performance liquid chromatography (HPLC), gradient grade, ≥99.9%), poly(ethylene glycol) methyl ether methacrylate (average *M_n_* = 500 Da, PEGMA500), poly(ethylene glycol) methyl ether methacrylate (average *M_n_* = 300 Da, PEGMA300), sodium acetate (≥99%), 4-((((2-carboxyethyl)thio)carbonothioyl)thio)-4-cyanopentanoic acid (CTA, 95%), lithium phenyl-2,4,6-trimethylbenzoylphosphinate (LPTP, ≥95%), deuterium oxide (D_2_O), deuterium chloride (DCl), phosphate-buffered saline (PBS), 2,2,2-trifluoroethanol (TFE, ≥99%), sodium trifluoroacetate (NaTFAc, ≥98%), Proteinase K from *Tritirachium album*, sodium chloride (≥99%), low-endotoxin bovine serum albumin (BSA), Triton X-100, and melittin were purchased from Sigma-Aldrich. The cell viability reagent alamarBlue, lysogeny broth (LB)-Lennox, and RPMI 1640 medium with and without phenol red were purchased from Thermo Fisher.

### Instrumentation

Reverse-phase analytical high-performance liquid chromatography (HPLC) was performed at 35 °C with a flow rate of 1 mL/min on an Alliance system from Waters equipped with an XBridge C18 column (4.6 × 50 mm^2^, 3 μm) and a photodiode array detector (Waters 2489 ultraviolet (UV)–visible) to assess the purity of peptides and conjugates, as well as to monitor the progress of conjugation reactions and dialysis. Preparative HPLC, using a C18 column (30 × 150 mm^2^, 5 μm) at room temperature with a flow rate of 25.52 mL/min and a photodiode array detector (Waters 2489 UV–visible), was used to purify crude peptides and conjugates. Samples were prepared in HPLC solvent (5% v/v ACN/water with 0.1% v/v TFA).

Nuclear Magnetic Resonance (NMR) spectroscopy was conducted on a 400 MHz Burke Neo NanoBay NMR spectrometer in D_2_O. Chemical shifts were referenced to the solvent residual peak at 4.79 ppm. Spectra were analyzed with MestReNova v14.3.2–32681.

Matrix-assisted laser desorption/ionization-time-of-flight (MALDI-TOF) mass spectrometry (MS) was performed on a Shimadzu MALDI-8030 system with a 200 Hz solid-state laser (355 nm). The instrument was calibrated with a standard MALDI calibration kit (TOFMix by Shimadzu, 670 fm/μL in 70% v/v ACN with 0.1% v/v TFA, tolerance: 450 mg/mol). Samples (1 μL, 1 mg/mL in HPLC solvent) were coated with an α-cyano-4-hydroxycinnamic acid (CHCA) matrix (1 μL, 5 mg/mL in 70% v/v ACN with 0.1% v/v TFA) on a stainless-steel plate and thoroughly dried in air before measurement.

Size exclusion chromatography (SEC) was performed in TFE with 0.02 M NaTFAc at a rate of 0.3 mL/min using a Tosoh system equipped with two isocratic pumps (one for the sample, the second for the solvent reference), a degasser, an autosampler, one 4.6 × 35 mm^2^ TSKgel guard super AW-H column (bead diameter: 9 μm), two 6 × 150 mm^2^ TSKgel super AWM-H linear analytical columns (beads diameter: 9 μm), and a refractive index detector. Number-average molecular weight (*M*_*n*_) and dispersity (*Đ*) were determined relative to poly(methyl methacrylate) (PMMA) standards. Samples were prepared at 2–3 mg/mL with an injection volume of 20 μL.

### Peptide Synthesis, Deprotection, and Purification

The prestapled peptide for copolymerization, Fmoc-Ahx-PESKAIKA(pentenyl)LLKA(pentenyl)VSKERSKRSP-NH_2_, was synthesized using a CEM Liberty Blue microwave-assisted solid-phase peptide synthesizer as described previously.^27^ The stapling reaction was conducted via a ring-closing metathesis reaction on resin between the alkene groups of the pentenyl alanine residues in a 10 mM solution of Grubbs first-generation catalyst in DCE. The Fmoc group was removed by 20% v/v piperidine in DMF after the stapling reaction was finished, followed by capping with methacrylic anhydride to prevent side reactions during the stapling procedure involving either a primary amine or methacrylamide with Grubbs’ catalyst^29^ with pentenyl alanine residues. The peptide-loaded resin was washed with DCE to remove DMF before the addition of the catalyst solution. The catalyst solution was deoxygenated with bubbling N_2(g)_ at room temperature for 20 min and then added to the resin-bound peptide in a reaction vessel to react for 30 min at 40 °C. The mixture was drained, and the peptide-bound resin was washed with DCE and then recharged with Grubbs catalyst solution under the same conditions as above. The mixture was again drained and washed with DCE. Following the stapling reaction, the N-terminus Fmoc-group next to Ahx was removed with 20% v/v piperidine in DMF to reveal the amine group. The resin-bound stapled peptide was then capped with a polymerizable methacrylamide group by reaction with methacrylic anhydride (10% v/v in DMF) at 65 °C for 2 min 30 s. Peptides were then deprotected in an acidic cleavage cocktail (92.5% v/v TFA, 2.5% v/v TIPS, 2.5% v/v DODT, 2.5% v/v DI water) and purified by preparative HPLC. We noticed a higher purity and therefore a higher recovery (ca. 30%) out of preparative HPLC when using a synthesis scale of 0.1 mmol as compared with a larger scale of 0.25 mmol (ca. 15–20%). Synthesis and purification of the peptide were confirmed by MALDI-TOF MS (Figure S1). [M + Na]^+^: *m*/*z* calculated = 2712.7, found 2712.3.

### RAFT Copolymerization of PEGMA and Stapled P9 Monomer

The polymerization mixture was prepared by dissolving reagents in acetate buffer (pH = 5) in a 2 mL chromatography vial, with the reagent amounts for preparing each copolymer listed in Table 1. We note that the comb-like conjugates are named as “the target number of AMPs per chain - MW of PEG side chain (g/mol)” as “4–500”, “8–500”, “4–300”, “8–300”, and “16–300”. To prevent the lysine amines from cleaving the chain transfer agent, polymerizations were conducted in 1 M acetate buffer (pH = 5).^30^ The PEGMA monomer was purified by passage through an aluminum-packed column immediately before use. For polymerization, considering the large peptide monomer may reduce the polymerization rate, we chose a molar ratio of CTA:initiator = 1:0.3 to accelerate the reaction while maintaining control of chain length. For the synthesis of conjugates with 4 or 8 peptides per chain, the initiator concentration was held constant. For the synthesis of conjugates with 16 peptides per chain, we lowered the initiator concentration to ensure the lysine amines could still be protonated by the buffer since holding the initiator concentration constant would require doubling the peptide monomer concentration, and these higher concentrations of peptide amines may exceed the capacity of the acetate buffer. Considering that low amounts of CTA and initiator can be hard to measure, stock solutions (10 mg/mL) were prepared and added to the vial. The total volume was then corrected by adding acetate buffer to the target volume. Mixtures were deoxygenated with bubbling N_2(g)_ at room temperature for 15 min and then sealed with parafilm. Polymerization was conducted in a UV box (365 nm, Analytik Jena UVP Cross-linker, CL-3000L) for 5–7 h at room temperature.

**Table 1:** Reagent Amounts for RAFT Copolymerizations

	stapled P9 monomer		PEGMA		CTA		initiator (LPTP)	
	equiv	concentration mM (mg/mL)	equiv	concentration mM (mg/mL)	equiv	concentration mM (mg/mL)	equiv	concentration mM (mg/mL)
conjugate 4–500	4	14.3 (50)	20	71.4 (35.7)	1	3.6 (1.1)	0.3	1.1 (0.3)
conjugate 8–500	10	35.7 (125)	40	142.9 (71.4)	1	3.6 (1.1)	0.3	1.1 (0.3)
conjugate 4–300	4	14.3 (50)	20	71.4 (21.4)	1	3.6 (1.1)	0.3	1.1 (0.3)
conjugate 8–300	10	35.7 (125)	40	142.9 (42.8)	1	3.6 (1.1)	0.3	1.1 (0.3)
conjugate 16–300	24	42.9 (150)	80	142.9 (42.8)	1	1.8 (0.5)	0.3	0.5 (0.2)

For kinetics studies, the polymerization mixtures were prepared, divided into aliquots (120 μL) in separate vials, and deoxygenated separately by sparging with N_2(g)_. The vials were placed in the UV box together to start the polymerization at the same time and removed at different time points (*t* = 0, 0.5, 1, 2, 3, and 4 h) for analysis. After removal, the mixtures were exposed to air to quench the polymerization reaction, transferred into different 7 mL glass vials, and then lyophilized. The dried mixtures were dissolved in D_2_O with DCl (50 μL DCl in 20 mL D_2_O, 0.6 mL) for ^1^H NMR analysis (Figures S2–S4). We added DCl to lower the pD of the NMR sample and prevent aminolysis of CTA by the amine groups of lysine upon redissolving the mixtures in D_2_O. To calculate the conversion of PEGMA, we analyzed the ^1^H NMR spectra by setting the resonance of the proton on the PEGMA polymerizable group (6.2 ppm) to 1 and compared it to integrations of the PEG methoxy protons at 3.4 ppm at different time points. For the conversion of peptide monomers, we rescaled the integration on the NMR spectra by setting one of the protons on the peptide monomer polymerizable group (5.7 ppm) to 1 and compared it to integrations of the Lys εH resonances at 3.0 ppm at different time points. These polymerizations to gauge kinetics measurements were performed in triplicate.

To prepare the conjugates, we prepared polymerization mixtures as described in Table 1 with similar moles of PEGMA to target similar masses of the final products. We stopped the polymerizations after 5 h for conjugates 4–500 and 8–500, and after 6 h for conjugates 4–300 and 8–300, considering that the shorter, more hydrophobic 300 g/mol PEGMA might have a slower polymerization rate than the longer (500 g/mol) PEGMA. For the preparation of conjugate 16–300, since we lowered the concentration of radicals to prevent aminolysis of the CTA, we chose a longer polymerization time (7 h). The conversions of each copolymerization were determined from ^1^H NMR spectra (Figure S5) similarly as described for our kinetics analysis. Considering that stapled P9 could complicate the polymerization due to the presence of a radical-reactive double bond in the staple, we also compared the integration of protons on the staple to that of lysine εHs for each purified conjugate (Figure S6 and Table S1).

The reaction mixtures were lyophilized and redissolved in 10% v/v ACN in DI water with 0.1% v/v TFA for dialysis. We used ACN to break possible aggregates and added TFA to keep a low pH and prevent possible aminolysis. The reaction mixtures were dialyzed against the same solvent in a dialysis membrane with a molecular weight cutoff (MWCO) of 3 kDa in a 4 L glass beaker for a week with solvent changes every day to remove unreacted monomer and switch the counterions to TFA. Purified conjugates were lyophilized and stored at −20 °C until use. AMP content (wt %) was determined by the average number of AMPs per chain × (AMP MW/conjugate MW), with conjugate MW = CTA MW + DP_AMP_ × stapled P9 monomer MW + DP_PEGMA_ × PEGMA MW]. Yields were calculatedas the mass of the conjugates/(total mass of the monomers plus CTA),assuming full conversion of both monomers and accounting for excess peptide.

### Secondary Structure

Circular dichroism spectroscopy was run under N_2(g)_ in a 0.1 cm path length quartz cell at 25 °C using a JASCO (Easton, Maryland) J-1500 CD spectrophotometer with a Peltier thermostated single-position cell holder. Peptide and conjugate samples were prepared at concentrations of 200 μM peptide equivalents in 10 mM PBS (pH = 7.4). While we acquired spectra from 190 to 250 nm in triplicate (Figure S9), we noticed the signals became noisy between 190 and 195 nm, possibly due to the relatively high sample concentration, and therefore only show spectra from 195 to 250 nm in the main text.

### Size, Zeta Potential, and Morphology of the Peptides and the Corresponding Conjugates

Dynamic light scattering (DLS) and zeta potential measurements were performed on a Malvern Zetasizer Ultra with a 4.0 mW laser (633 nm) at 25 °C. Samples were prepared at concentrations of 200 μM peptide in filtered (0.45 μm pore) 10 mM PBS and adjusted to pH = 7.4 using NaOH and HCl. Both size and zeta potential were measured in 10 mM PBS to limit the salt concentration and permit the calculation of zeta potential from electrostatic mobility using the Smoluchowski approximation.^31^ Size measurements were performed in square DTS0012 cuvettes (Malvern) in triplicate. We used the *Z*-average diameters as the hydrodynamic diameters of the particles to capture the contributions of both single chains and larger structures. To validate the DLS results determined using the manufacturer’s software (ZS Xplorer 2.10), we fitted the correlograms using a nonlinear cumulant analysis^32^ with decay models including either one or multiple size distributions of the particles (Figures S10–S12). Zeta potential measurements were performed in DTS1070 cuvettes in triplicate and are reported as the mean ± the standard deviation (Table S3).

Transmission electron microscopy (TEM) was performed using an FEI Titan instrument operating at an accelerating voltage of 120 kV. Peptide and conjugate samples were normalized at peptide equivalent concentrations of 200 μM in 10 mM PBS (pH = 7.4). Carbon-coated copper grids (300 mesh, Electron Microscopy Sciences) were pretreated with 20% v/v O_2(g)_ and 80% v/v Ar_(g)_ in a plasma cleaner for 45 s. Samples (3 μL) were added to the grids for 1 min, blotted with filter paper by placing filter paper at the edge of the grid to remove excess solution and washed once by quickly dabbing and blotting off a drop of deionized (DI) water (10 μL). Washed grids were dried for 1 min before being stained with a 2% aqueous uranyl acetate solution (3 μL) for 1 min. Excess uranyl acetate solution was blotted off with filter paper and the samples were air-dried before imaging at magnifications ranging from 10,000 to 34,000× (Figures S13–S15).

### Proteolytic Stability of Stapled P9 and the Corresponding Conjugates

Solutions of Proteinase K (8 μM, 0.224 mg/mL) and the tested peptides/conjugates (400 μM peptide equivalent) were prepared in 1X PBS (pH = 7.4). Mass concentrations of stapled P9 and the conjugates (4-arm, 8-arm, 4–500, 8–500, 4–300, 8–300, and 16–300) were 1.34, 2.85, 2.51, 2.66, 2.31, 2.10, 2.00, and 1.98 mg/mL, respectively. Solutions of peptides/conjugates (0.6 mL) were mixed with a Proteinase K stock solution (0.6 mL) and filtered into a 2 mL HPLC sample vial for characterization. HPLC was used to monitor degradation by injecting sample mixtures (80 μL) after incubation in the presence or absence of Proteinase K for 0.5, 1, 2, and 24 h. Control samples were prepared by diluting the corresponding stock solutions (0.6 mL) with PBS alone (0.6 mL). The peak area integrations were integrated by Empower HPLC software (Figures S17–S24 and Tables S4 and S5).

### Antimicrobial Activity of Stapled P9 and Corresponding Conjugates

The antimicrobial activity of peptides, conjugates, and polymer control was evaluated against a well-characterized multidrug-resistant (MDR) clinical isolate,*Klebsiella pneumoniae* BL13802,^33,34^ using the metabolic indicator dye alamarBlue to quantify bacterial viability/survival, as previously described.^27,35^ Briefly, bacteria grown in LB medium to an OD_600_ of ∼0.6 were diluted in RPMI medium and combined in a 1:1 ratio with medium alone or containing peptides/conjugates in the wells of a 96-well plate (200 μL total volume with ∼2.5 × 10^5^ CFU/mL and final peptide equivalent concentrations of 200 or 100 μM). Wells containing only RPMI medium were included in each experiment as a sample blank. After exposure (2 h at 37 °C with shaking, 270 rpm), triplicates of all samples (50 μL per replicate) were replated into individual wells of a clear-bottom, black-wall microplate. An equal volume of 2× LB medium was then added to each well, followed by the alamarBlue reagent (10 μL). The sample plate was protected from light with foil and incubated at 37 °C (without shaking) until the untreated control bacteria, exposed to media alone, began to change color from blue to purple (approximately 2–3 h). At this point, the fluorescence intensity (excitation 530 nm; emission 580 nm) of each well was measured using a VICTOR Nivo multimode plate reader (PerkinElmer). We verified that the untreated bacteria wells, which are expected to give the highest signal, remained below 6 million relative fluorescence units (RFU) and therefore within the linear range of the instrument’s detector for this assay. To calculate percent bacterial survival, blank-corrected sample signals were divided by those obtained from the untreated control.

### Hemolysis of Stapled P9 and Corresponding Conjugates

Research utilizing human-derived materials was approved by the University of Virginia Institutional Review Board for Health Sciences Research (IRB-HSR; protocol #13909). Red blood cells (RBCs), isolated from Ficoll–Paque separations of human whole blood, were diluted 1:10 with PBS (pH 7.4) in a 50 mL centrifuge tube. The solution was mixed by inversion and centrifuged at 500*g* for 10 min at room temperature. RBCs were washed twice more with PBS as above and then diluted 1:20 (v/v, 5% final) in RPMI medium (without phenol red) that was supplemented with low-endotoxin BSA (1% final). Medium lacking phenol red was used since the color imparted by this pH indicator interferes with the absorbance measurement that quantifies hemolysis. Processed RBCs (180 μL) were combined with test peptides/conjugates (20 μL, 2 mM peptide equivalent) in triplicate in the wells of a round-bottom microplate. Included in each assay were also samples containing RBCs together with the hemolytic peptide melittin (positive control), media alone (negative control and analytical blank), H_2_O (vehicle control), or Triton X-100 (1% final; complete hemolysis). Following incubation (1 h at 37 °C), the assay plate was centrifuged at 500*g* for 5 min. Supernatants (100 μL) were transferred to a fresh flat-bottom microplate, and sample absorbance was measured at 540 nm using a VICTOR Nivo plate reader. To calculate percent hemolysis, blanked sample absorbance readings were divided by those obtained from RBCs exposed to Triton X-100.

## Results and Discussion

### Design and Synthesis of Comb-Like Stapled P9-PEG Conjugates

**Figure 1:**
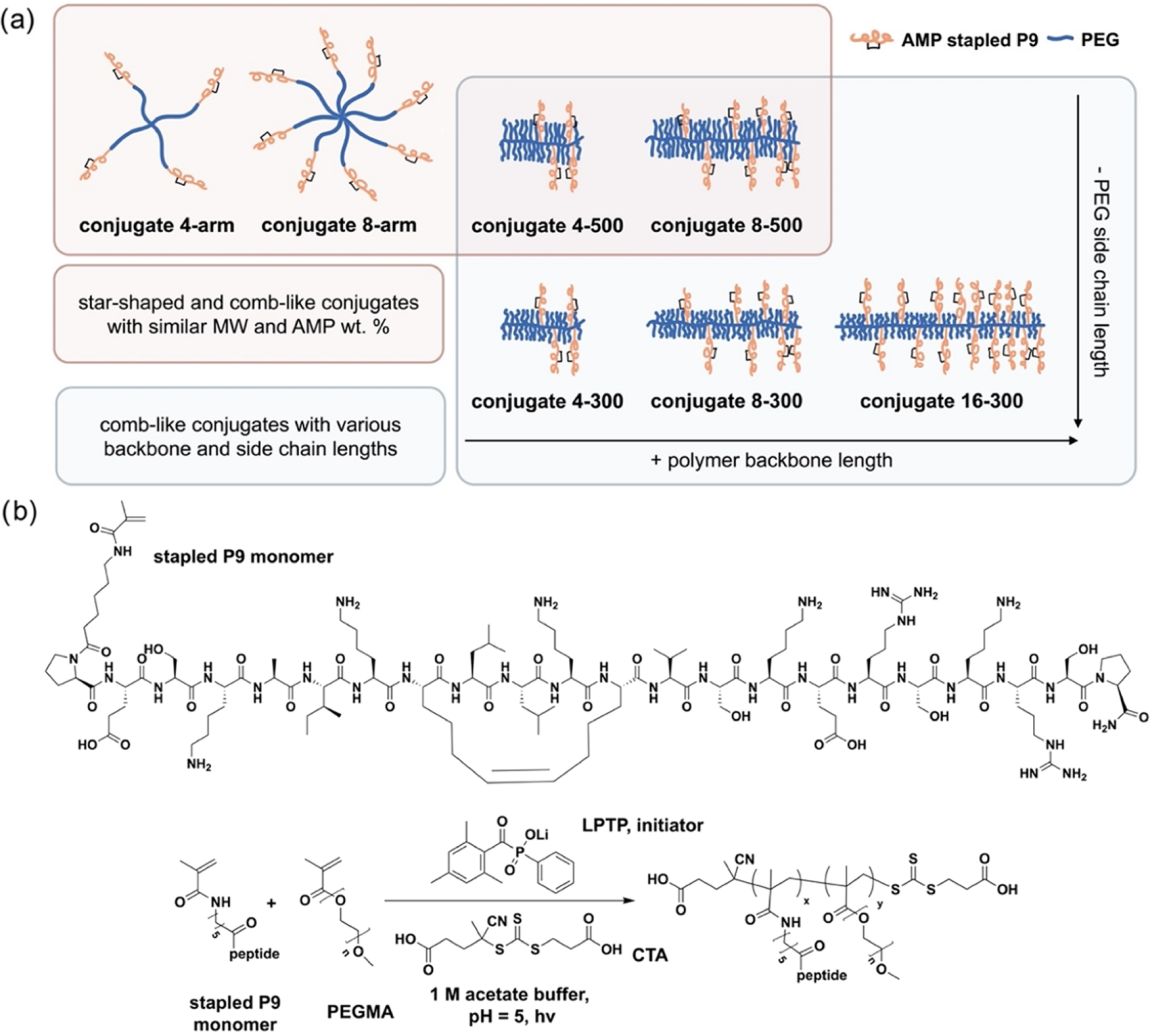
Design and synthesis of star-shaped and comb-like conjugates of PEG and the AMP, stapled P9. (a) Design space and nomenclature of the star-shaped and comb-like conjugates in this work. Star-shaped conjugates are named by “the number of AMP arms” as “4-arm” and “8-arm”.^26^ Comb-like conjugates are named as “the target number of AMPs per chain - MW of PEG side chain (g/mol)” as “4–500”, “8–500”, “4–300”, “8–300”, and “16–300”. Two pairs of conjugates, 4–500 and 4-arm, and 8–500 and 8-arm have similar MW, peptide wt %, and number of AMPs that allowed us to compare star-shaped and comb-like architectures. (b) Structure of the stapled P9 monomer (top) and copolymerization of stapled P9 monomer with PEG monomer to prepare comb-like conjugates (bottom).

To understand the role of polymer architecture (comb-like vs. star-shaped) as well as the effects of PEG side chain length and backbone length of the comb-like polymers, we designed a series of comb-like conjugates varying in the target number of AMPs per chain (4, 8, or 16) and the molecular weight (MW) of the PEG side chains (300 or 500 g/mol) (Figure 1a). The naming system we employ includes both of these variables: number of AMPs per chain—PEG MW. For example, a conjugate prepared by targeting 16 AMPs per chain and with PEG side chains with MW of 300 g/mol is called 16–300. As the AMP component of our conjugates in this study, we selected the sequence used in our previous work, stapled P9, which is derived from the C-terminal, helical antimicrobial domain of the human chemokine CXCL10.^27,35^

For the comparison of comb-like vs star-shaped architectures, we designed comb-like conjugates 4–500 and 8–500 to match the compositions (wt % peptide) and molecular weights of our previously reported 4- and 8-arm star-shaped conjugates with the same AMP.^27^ Then, to understand the effect of PEG side chain length on the solution properties and antimicrobial performance of comb-like conjugates, we designed conjugates 4–300 and 8–300 with similar backbone degree of polymerization (DP) to conjugates 4–500 and 8–500, respectively. Finally, we varied polymer backbone length by synthesizing conjugate 16–300 with similar mol % AMP, but with a higher backbone DP than conjugates 4–300 and 8–300.

Comb-like conjugates were synthesized by photoinitiated RAFT copolymerization of AMP- and PEG-functionalized monomers in aqueous acetate buffer. The AMP-functionalized monomer features the AMP stapled P9 connected to a polymerizable methacrylamide group via an aminohexanoic (Ahx) linker to space the N-terminal proline residue away from the polymerization site and reduce steric hindrance (Figures 1b and S1). The PEG-functionalized monomers include 300 and 500 g/mol PEG methacrylate (PEGMA). We selected aqueous acetate buffer (pH 5) to avoid aminolysis of the chain transfer agent. An initial polymerization showed the PEG monomers to incorporate faster than the larger peptide monomers, with essentially quantitative conversion of PEGMA and just 50% conversion of stapled P9 monomer after 3 h (Figures S2–S4). The slower conversion of the AMP monomer relative to PEGMA suggests that the AMPs do not distribute evenly along the polymer backbone, but in a gradient, with a higher density of AMPs at the end of the copolymer chain. While similar monomer incorporation rates are ideal for controlling sequence, the gradient distribution may lead to high local densities of the peptide that may benefit activity^25,26^ and stability^36^ – an interesting aspect deserving of further attention, but one that is beyond the scope of this manuscript. To balance the polymerization rate difference between the peptide monomer and PEGMA and reach the target number of peptides per chain, we fed excess peptide monomer according to the conversion we observed in polymerizations without excess peptide (Table 1). We determined the conversion of both monomers and calculated the AMP content (i.e., AMP wt %) of each conjugate using ^1^H NMR spectra acquired at the end of the copolymerizations (Figures S5, 6S and 2a, and Table S1). Syntheses of conjugates 4–500 and 8–500 achieved satisfyingly similar MW and AMP content (43 and 51 wt %, respectively) as their star-shaped analogs (44 and 51 wt %, respectively^27^), and therefore, are well suited for making comparisons across comb-like and star-shaped architectures. Additionally, we obtained comb-like conjugates 4–300, 8–300, and 16–300 with a constant AMP content of ca. 60 wt % to study the effects of polymer backbone length. For characterization of the solution properties of all conjugates, a peptide equivalent concentration of 200 μM was selected to yield a mass concentration of ca. 1–1.5 mg conjugate/mL in PBS, suitable for circular dichroism spectroscopy, dynamic light scattering, zeta potential, and transmission electron microscopy (TEM) measurements we use to probe secondary structure, size, morphology, and surface charge of the conjugates.

**Figure 2:**
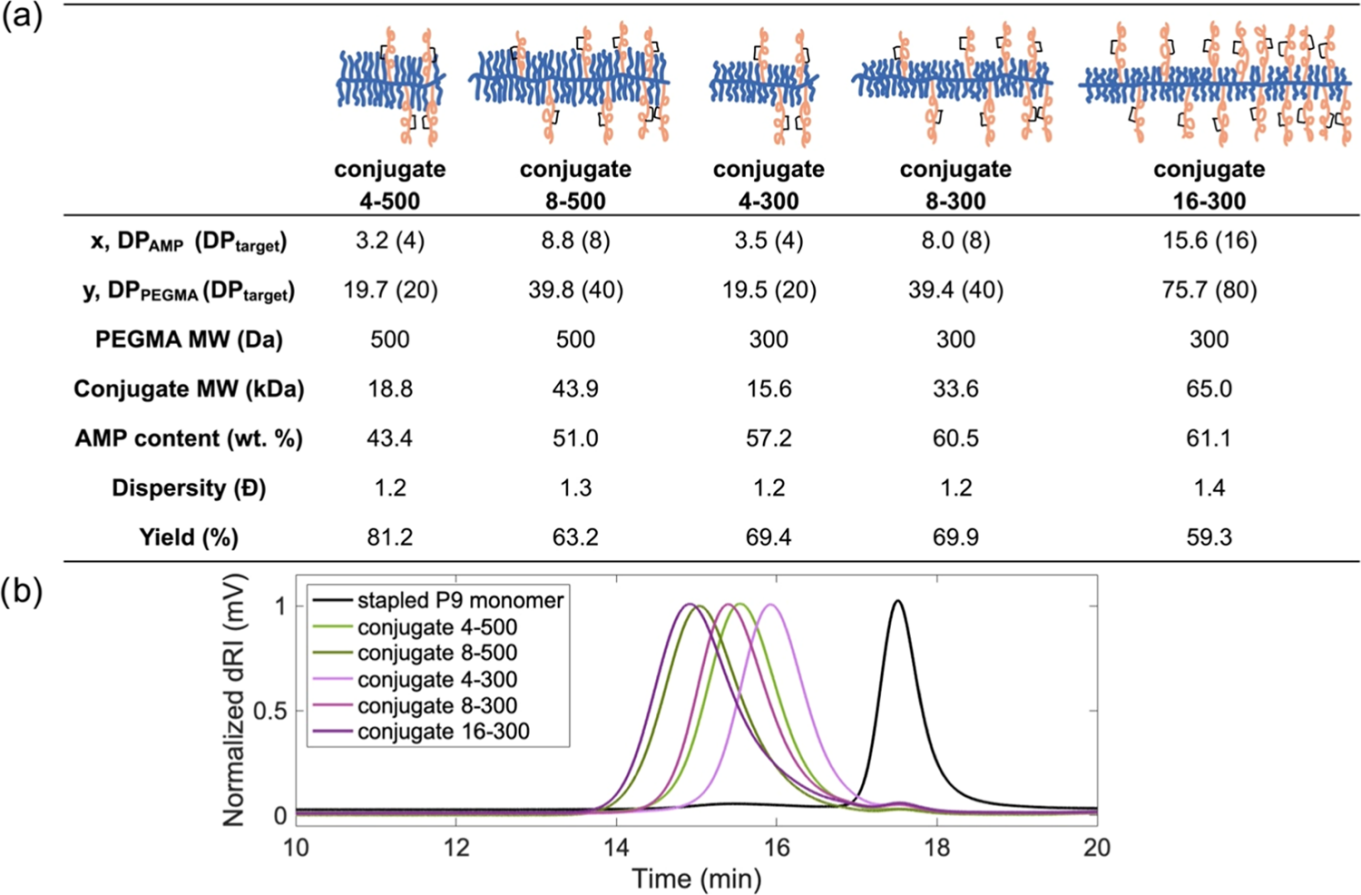
Comb-like stapled P9-PEG conjugates: (a) composition, dispersity, and yield of the conjugates. DP_AMP_ and DP_PEGMA_ were determined by ^1^H NMR (Figures S5 and S6). Conjugate MW was calculated: as [CTA MW + DP_AMP_ × stapled P9 monomer MW + DP_PEGMA_ × PEGMA MW]. AMP content (wt %) was determined by the average number of stapled P9 per chain × AMP MW/conjugate MW. Yields were calculated assuming full conversion of both monomers, as the mass of the conjugates/total mass of the monomers and CTA. (b) SEC traces of the peptide monomer and the comb-like conjugates (1–3 mg/mL in trifluoroethanol).

### Secondary Structure

**Figure 3:**
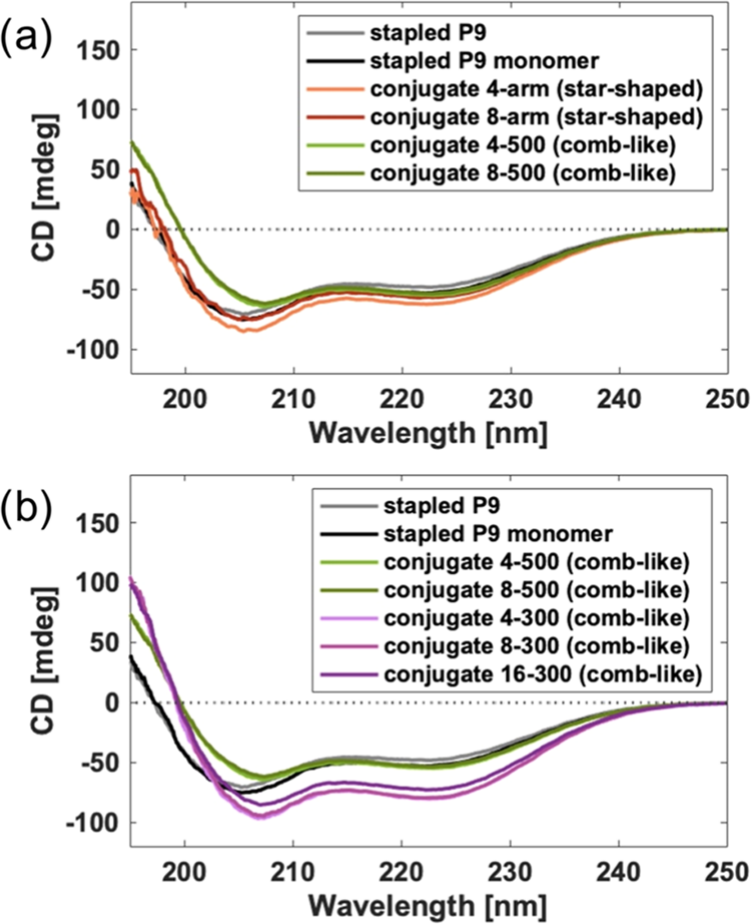
CD spectra: (a) Free peptides and the corresponding star-shaped and comb-like conjugates with similar compositions; (b) Free peptides and comb-like conjugates. Measurements were taken at 200 μM peptide equivalents in 10 mM PBS (pH = 7.4).

Given that the secondary structure of an AMP, and in particular the helicity, is known to impact antimicrobial activity,^35^ we first characterized the helicity of the AMPs on the conjugates by circular dichroism (CD) spectroscopy. As shown in Figure 3a, the spectra of both star-shaped and comb-like conjugates display characteristic features of α-helical peptides (λ_min_ at 190–195, 205–208, and 222–224 nm) similar to unconjugated stapled P9 and stapled P9 monomer, suggesting that connecting the peptide to polymers does not disrupt its α-helical secondary structure (Figure 3a). While both star-shaped and comb-like conjugate spectra featured similar intensities of the minimum at 222–224 nm, the comb-like conjugates have a minimum at 208 nm and a higher intensity 195 nm peak compared to those of the free peptides and the star-shaped conjugates with a minimum at ca. 205 nm with lower signal at 195 nm. These differences were similar to our previous observations when comparing the spectra of the star-shaped conjugates in buffer and the helix-promoting solvent trifluoroethanol (TFE), suggesting that peptides on the comb-like conjugates are more helical than those on star-shaped materials.^27^ We speculate this enhancement in helicity of comb-like conjugates relative to either their star-shaped analogs or unconjugated AMPs is due to either a locally more hydrophobic environment or an increase in the local peptide concentration.^37,38^

Among the comb-like conjugates (Figure 3b), those with the same side chain lengths, but different backbone lengths, did not show significant differences in helicity. Yet, conjugates with shorter PEG side chains (i.e., 300 vs. 500 g/mol) showed more helical character with higher signals at both 208 and 224 nm than those with longer side chains. A possible explanation is that the shorter PEG side chains are more hydrophobic^28^ and may not detract as much from intermolecular peptide hydrogen bonding that stabilizes helices. While shortening PEG side chains on comb-like conjugates increased helicity, our previous work showed that shortening PEG arms on star-shaped conjugates did not.^27^

### Solution Behavior

**Figure 4:**
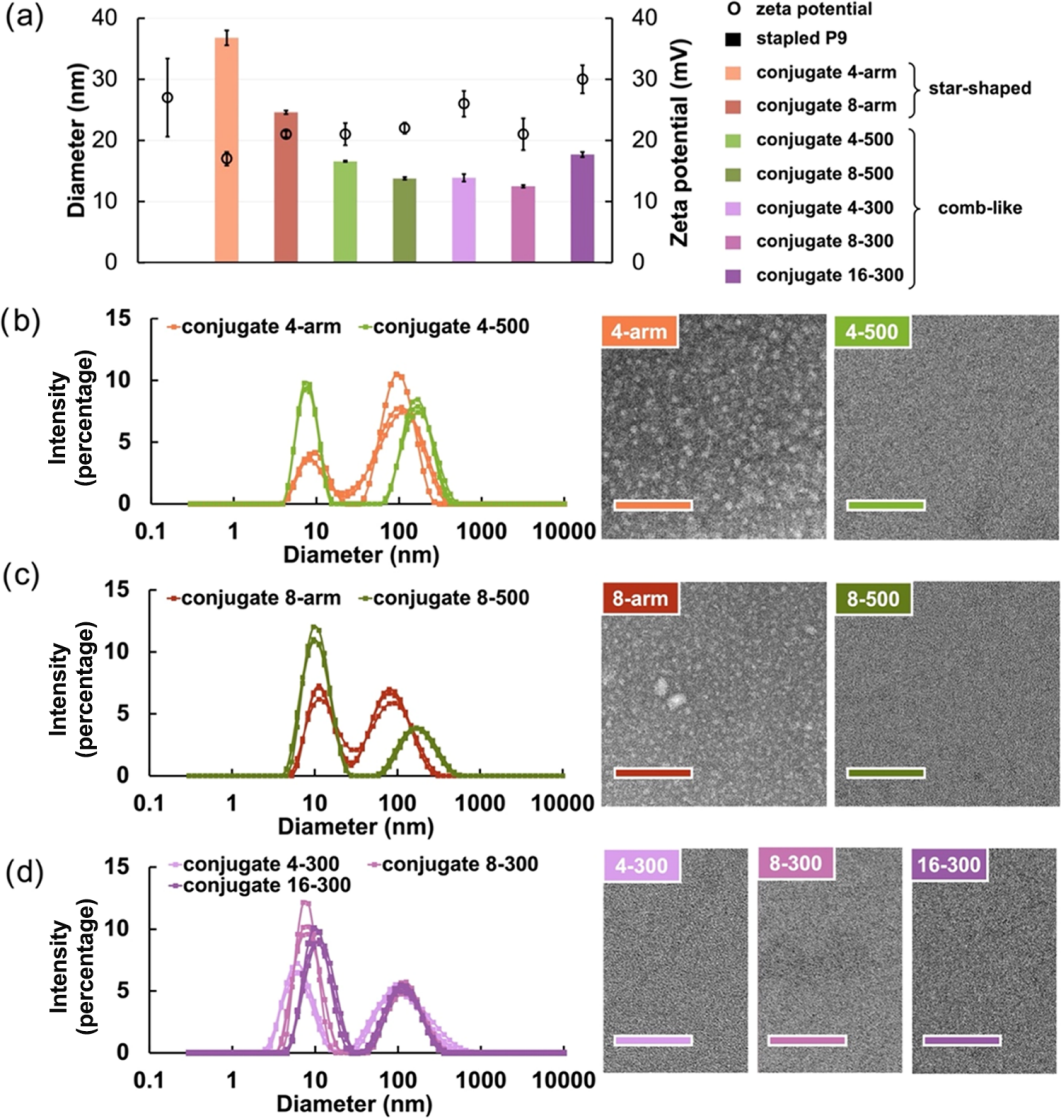
Size, zeta potential, and morphology of stapled P9 and the conjugates as a function of molecular architecture (comb-like vs. star-shaped), polymer backbone length, and side chain length: (a) size and zeta potential data from samples at 200 μM peptide equivalent concentrations in 10 mM PBS (pH = 7.4); (b) DLS intensity profiles and TEM images of comb-like conjugate 4–500 (green) and the analogous star conjugate (4-arm, orange); (c) DLS intensity profiles and TEM images of comb-like conjugate 8–500 (olive) and the analogous star-shaped conjugate 8-arm (red); (d) DLS intensity profiles and TEM images of comb-like conjugates varying in polymer chain length: 4–300 (light purple), 8–300 (purple), and 16–300 (dark purple). All DLS and zeta potential measurements were acquired in triplicate, with error bars representing standard deviation. Scale bars: 100 nm.

To understand how the comb-like vs star-shaped conjugates and comb-like conjugates with different PEG side chain lengths and backbone lengths present AMPs in solution, we measured the zeta potential and the *z*-average hydrodynamic diameter of the conjugates in 10 mM PBS at a constant peptide equivalent concentration (200 μM) and related these results to TEM images (Figure 4).

All conjugates showed comparable or just slightly reduced zeta potentials relative to the unconjugated AMP (+27 mV) (Figure 4a), suggesting that these nonlinear polymers do not shield the AMPs, but avail them for electrostatic interactions with bacterial membranes. Comb-like conjugates 4–500 and 8–500 had comparable zeta potentials (ca. + 21 mV) relative to their star-shaped analogs (+17 and 21 mV, respectively). With respect to PEG side chain length, conjugate 4–300 showed a slightly higher zeta potential (+26 mV) than conjugate 4–500 (+21 mV), suggesting that conjugates with shorter PEG side chains render AMPs more accessible in solution due to less steric hindrance. This observation is consistent with a previous study on DNA-PEG bottlebrush conjugates,^38^ which showed shorter PEG side chains to result in a higher surface charge (more negative zeta potential). However, conjugates 8–300 and 8–500 have similar zeta potentials, possibly because the NMR spectra show that despite targeting and achieving ca. 8 AMPs per chain, conjugate 8–500 has a slightly higher number of cationic AMPs (8.8 per chain) than 8–300 (8 per chain) (Figure 2a). Increasing backbone length at constant AMP density and PEG side chain length led to the highest zeta potential (+30 mV for conjugate 16–300) among the comb-like conjugates, presumably due to these conjugates presenting the highest number of AMPs per chain and therefore featuring the highest local concentration of AMPs.

Regarding hydrodynamic diameter, all comb-like and star-shaped conjugates showed two size distributions, one attributed to single chains and the other to supramolecular assemblies or aggregates (Figures 4b,c and S10–S12, and Table S3). The Z-average hydrodynamic diameter we calculated from the DLS measurements accounts for both of these populations, though we also note the percentage of scattering intensity from single chain vs larger structures when comparing the solution behavior of the various conjugates. Regardless of PEG side chain length and backbone length, all 5 comb-like conjugates show small (and similar) *Z*-average hydrodynamic diameters ranging from 13 to 18 nm (Figure 4a–d) with a higher percentage of scattering from single chain-sized structures. Between comb-like and star-shaped conjugates, the stars have larger *Z*-average hydrodynamic diameters (37 and 24 nm, respectively) than their comb-like analogs 4–500 and 8–500 (17 and 14 nm, respectively) (Figure 4a), consistent with the comb-like conjugates having a lower percentage of scattering intensity associated with larger structures (Figure 4b,c).

Corroborating this result, TEM images of the star-shaped conjugates show nanostructures, whereas images of the comb-like conjugates following the same sample preparation procedure are featureless, suggesting that the comb-like conjugates may not remain on the grids after three water washes (Figures 4 and S13). Reducing water washes from three to one revealed loosely resolved spherical structures from the comb-like conjugates (Figures S14 and S15), although it is possible these result from drying on the grid and do not reflect the solution morphology. The TEM results showing that the comb-like conjugates are more soluble and have lower propensity for supramolecular assembly than the star-shaped conjugates are consistent with the DLS size intensity profiles. These results are also consistent with coarse-grained molecular simulations^39^ showing comb-like (bottle-brush) polymers with solvophobic components to assemble less readily than analogous star-shaped polymers, attributed to the difficulty of the solvophobic components on adjacent copolymers to contact one another. We next sought to understand how the solution properties of comb-like and star-shaped conjugates played out in their resistance to proteolytic stability, antimicrobial activity, and compatibility with mammalian cells.

### Protease Resistance

**Figure 5:**
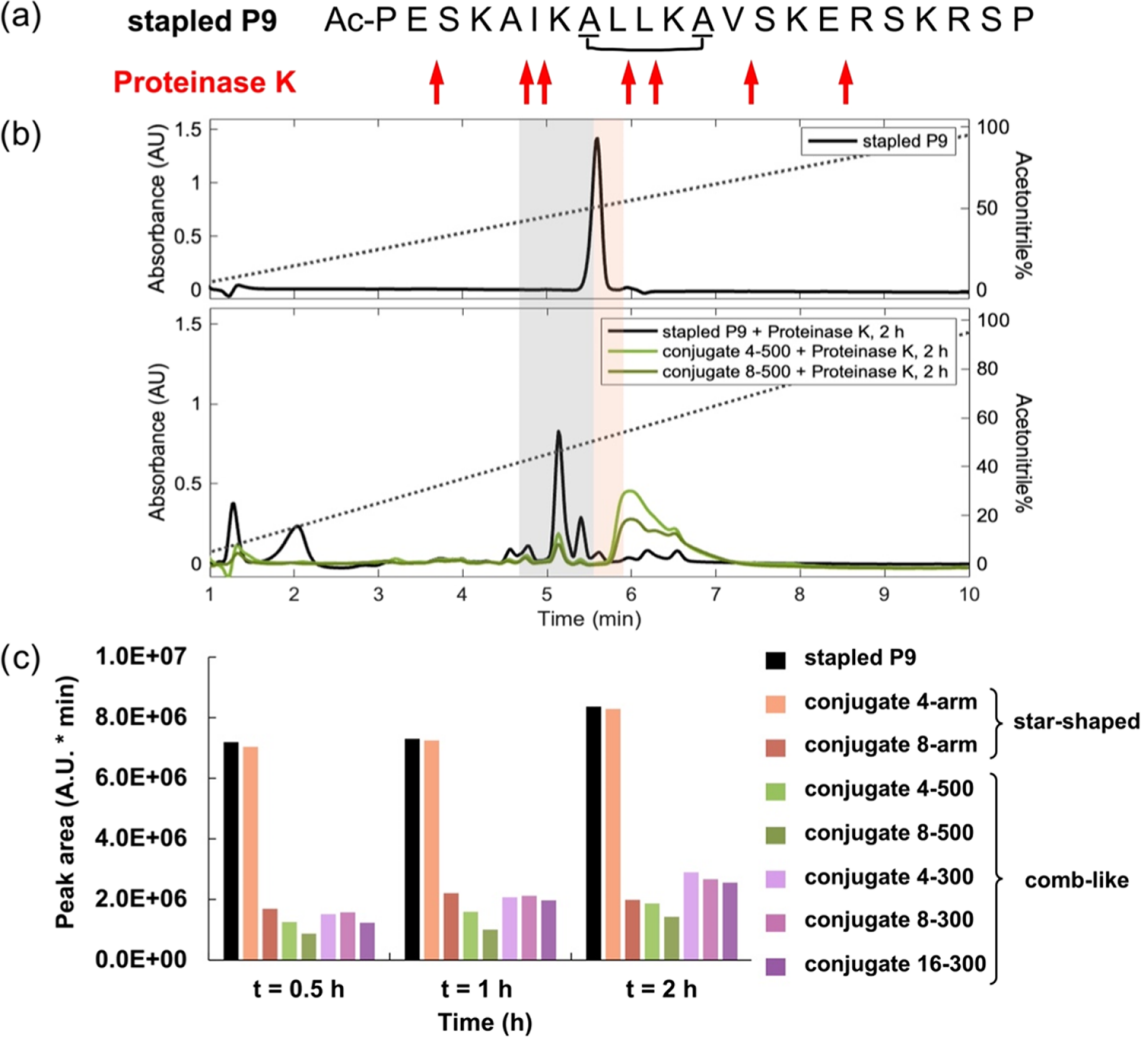
Stability of stapled P9 and conjugates in the presence of Proteinase K in 1X PBS. (a) The primary amino acid sequence of stapled peptide P9 with predicted cleavage sites that are indicated by red arrows (sites identified using Peptide Cutter). (b) HPLC trace of stapled P9 (highlighted in orange) and Proteinase K treated stapled P9, conjugates 4–500, and conjugate 8–500 for 2 h. The region highlighted in gray represents the degraded peptide fragments. (c) Peak areas of the peptide fragments after incubation with Proteinase K for 0.5, 1, and 2 h.

To probe the role of conjugate architecture and comb-like conjugate backbone length and PEG side chain length on the proteolytic stability of the AMP constituents, we selected Proteinase K as a model protease, since it is predicted to cleave stapled P9 at multiple sites.^27,40^ We compared the degradation rates of the unconjugated AMP to those appended to the conjugate materials using HPLC (Figures 5 and S17–S24 and Tables S4 and S5). Rather than monitor the disappearance of the intact conjugates, whose retention time and extinction coefficient may vary during degradation, we opted to monitor the appearance of degradation products eluting between 4.5 and 5.5 min. Since these degradation products further degrade, as judged by multiple peak shifts and a decrease in peak area in this region between 2 and 24 h (Figure S17), we compared the peak area of released peptide fragments after 2 h of protease treatment as an indication of the degree of degradation (Figure 5c).

The HPLC traces of all comb-like conjugates after incubation with Proteinase K showed a substantially slower appearance of the degraded peptide fragments compared to either the free peptide or the 4-arm star-shaped conjugate (Figure 5c). While the 8-arm star-shaped conjugate traces showed lower signal from the degraded peptide fragments, this may not necessarily be interpreted as superior proteolytic stability: we noticed the 8-arm star-shaped conjugate was just marginally soluble at the test concentration (1.2 mg/mL) in 1X PBS, which may have reduced the amount injected onto the HPLC after filtration and led to the lower peptide fragment signals (Tables S4 and S5). The various comb-like conjugates appear to release peptide fragments at similar, and markedly slower rates than the unconjugated peptide or 4-arm star-shaped conjugate. While not necessarily significant, the two comb-like conjugates with longer PEG arms (500 g/mol, green bars) show slightly lower peptide fragment signals than all three comb-like conjugates with short PEG arms (300 g/mol, purple bars), both at 2 h Figure 5c and after 24 h (Table S4). These data suggest the longer PEG chains may better shield the AMPs from the protease. Together, these data indicate that the comb-like architecture better protects the peptide from degradation.

Since star-shaped conjugates form larger structures in solution than the comb-shaped conjugates, one might reasonably expect the star-shaped conjugates to better protect the AMPs, yet we observe the opposite. One possibility is that the superior stability comes from a high-density presentation of peptides on the comb-like conjugates, consistent with a previous study on comb/brush peptide–polymer conjugates.^36^ Supporting this notion, the larger size of the star-shaped conjugates compared to the comb-like conjugates, considered together with the similar positive zeta potentials measured for both architectures, suggests that the star-shaped conjugates have lower local concentrations of AMPs than the comb-like conjugates.

### Antimicrobial Activity against *K. pneumoniae*

**Figure 6:**
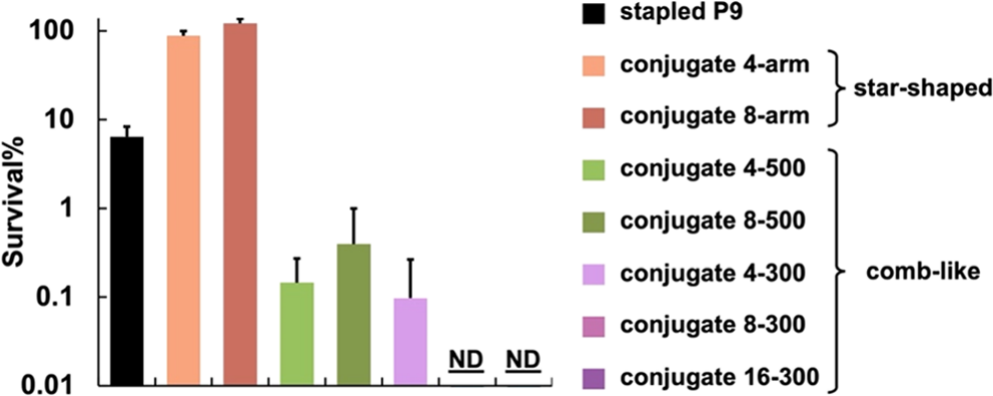
Bacterial survival (%) after treatment with the indicated AMP or conjugate (peptide equivalent concentration = 100 μM in RPMI medium). The assays were performed in triplicate. Results are plotted for each batch and the bars indicate the average. Error bars represent the standard deviation. ND: none detected (<0.01%).

To evaluate the bactericidal activity of the various conjugates, we used an alamarBlue viability assay employing multidrug-resistant *K. pneumoniae*. In this assay, metabolically active bacteria reduce the blue alamarBlue reagent to a pink fluorescent product. To obtain the percentage of metabolically active bacteria, we normalized the fluorescent signal generated from each sample to that produced by untreated bacteria. We tested two peptide equivalent concentrations (i.e., 100 and 200 μM) that best highlight differences in activity among the conjugates (Figure S25). Whereas unconjugated stapled P9 killed 92% of bacteria at 100 μM peptide, all comb-like conjugates killed >99% bacteria at the same peptide-equivalent concentration after 2 h (Figure 6). To further challenge these treatments, we incubated the samples overnight to give surviving bacteria time to replenish. Most cultures treated with the comb-like conjugates started to show pink products indicative of bacterial regrowth, yet no significant color change was observed from bacteria treated with conjugate 16–300 (Figure S26), suggesting superior killing activity by 16–300 than the other comb-like conjugates. In contrast, neither star-shaped conjugate showed bactericidal effects at 100 μM peptide equivalent concentrations even after 2 h. We attribute the superior antimicrobial activity of the comb-like conjugates to their effective presentation and protection of the AMPs, as well as the greater AMP helicity promoted by this architecture. To ensure that the antimicrobial activity comes from the AMPs and not the polymer alone, we evaluated the potential antimicrobial effects of a comb polymer control consisting of just the PEGMA repeating units (Table S2 and Figures S7, S8 and S27). The control polymer, with the same PEGMA DP as the most active 16–300 comb-like conjugate, did not kill bacteria after 2 h, allowing us to attribute the antimicrobial activity of the conjugates to the constituent AMPs and how they are displayed on the polymers.

### Hemolysis

Given that the comb-like conjugates were so much more active than their star-shaped analogs against bacteria, it was important to ensure that they were not also more toxic to mammalian cells. Therefore, we measured human red blood cell (RBC) hemolysis caused by the free peptide and conjugates at a constant peptide concentration of 200 μM. Neither the free peptide nor any conjugate caused significant hemolysis under the conditions examined (<5%; Figure S28), indicating that the comb-like conjugates, despite their high local concentrations of AMPs, did not introduce hemolytic activity.

## Conclusions

In summary, we showed comb-like architectures to be resoundingly superior to their star-shaped analogs at presenting the helical AMP stapled P9 in ways that maximize antimicrobial activity, solubility, helicity, and stability without compromising compatibility with mammalian cells. Moreover, the design flexibility inherent to comb architectures allowed us to design and synthesize comb-like conjugates that (a) match the AMP wt % and molecular weight of star-shaped conjugates we synthesized previously and (b) vary in PEG-side chain length and backbone length. Shortening the side chain length from more hydrophilic 500 g/mol to the more hydrophobic 300 g/mol PEG increased AMP helicity, although stability data suggest the shorter chains may not shield the AMP as well from proteases.

Relative to star-shaped conjugates, the comb-like conjugates were more soluble and less prone to supramolecular assembly. We saw this first in characterizing solution behavior, where DLS intensity profiles showed the comb-like conjugates to have a higher percentage of single chain-sized structures than the star-shaped conjugates and TEM showed no evidence of structures for comb-like conjugates following the same 3-water wash procedure as for star-shaped conjugates. Further supporting this notion is the lower solubility of the 8-arm star-shaped conjugate relative to the analogous comb-like conjugate at the high concentrations used for proteolytic stability measurements. While consistent with coarse-grained simulations showing bottlebrush polymers to be less prone to supramolecular assembly than analogous star-shaped polymers,^39^ there are limited comparisons of the assembly of comb-like polymers to star-shaped polymers with comparable compositions and molecular weights. We hope that our findings may motivate further investigation of the effects of different nonlinear polymer architectures on solution behavior and functionality.

Considering reasons for the superior performance of the comb-like architectures to their star-shape analogs sheds light on several important future directions. One possibility is that the lower propensity of the comb-like conjugates for supramolecular assembly leaves AMPs to interact with bacterial membranes unencumbered by supramolecular assembly. Continuing to investigate solution properties with antimicrobial performance will allow us to understand the undoubtedly important connection between the two. Another possibility is that the gradient distribution we observed of AMPs on comb-like conjugates, due to the faster incorporation of PEG relative to the larger AMP-functionalized monomers, may have resulted in segments of locally higher densities of AMP on the comb-like conjugates that make them more potent. Future studies involving modeling and controlling AMP monomer distribution within comb polymers would be incredibly valuable to parse out the effects of gradient vs. uniform AMP distribution. In addition to control of monomer distribution, comb-like architectures offer inherently large design spaces, since they are prepared by controlled polymerizations compatible with an expansive range of monomer chemistries that enable tailoring of peptide densities, distributions, and backbone lengths in ways that maximize function. Going forward, we will look to capitalize on the superior performance of the comb-like conjugates together with their design flexibility to deepen our understanding of how to present AMPs on polymers and accelerate their clinical implementation as alternatives to conventional antibiotics that are urgently needed to combat infections caused by multidrug-resistant bacteria.

## Supporting Information Available

Materials; experimental details; peptide and conjugate characterization; additional circular dichroism spectroscopy; dynamic light scattering; transmission electron microscopy; HPLC chromatograms for assessing proteolytic stability; antimicrobial activity data; and hemolysis data (PDF)
