# Supplementary material for: Presenting Antimicrobial Peptides on Poly(ethylene glycol): Star-Shaped vs Comb-Like Architectures
Source: Macromolecules. 2025 Feb 5;58(4):2073–84. doi: 10.1021/acs.macromol.4c02762 (PMC11867009; doi:10.1021/acs.macromol.4c02762)
Supplement: Supplementary file 1 — ma4c02762_si_001.pdf [file ma4c02762_si_001.pdf]

Supporting information: Presenting antimicrobial peptides on poly(ethylene glycol): star-shaped vs comb-like architectures

Zixian Cui[1], Elliot A. Brna[1], Matthew A. Crawford[2], Puthayalai Treerat[2], Mobina Alimadad[1], Molly A. Hughes[2], and Rachel A. Letteri[1]*

[1]Department of Chemical Engineering, University of Virginia, Charlottesville, VA 22903 USA

[2]Division of Infectious Diseases & International Health, Department of Medicine, University of Virginia, Charlottesville, VA 22908 USA

*Corresponding author: Rachel A. Letteri (rl2qm@virginia.edu)

Table of content

Peptide synthesis ---------- 2

^{1}H Nuclear magnetic resonance (NMR) spectra of stapled P9 monomer and PEGMA monomers ---------- 3

Kinetics of copolymerization of stapled P9 monomer and PEGMA500 ---------- 4

^{1}H NMR spectra of copolymerization mixtures ---------- 7

^{1}H NMR spectra of purified conjugates ---------- 8

Synthesis and characterization of conjugate 16-300 analogous polymer control -- 9

Circular dichroism (CD) spectra of stapled P9, stapled P9 monomer, and the comb-like conjugates ---------- 12

Dynamic light scattering (DLS) results of stapled P9 and the comb-like conjugates ---------- 13

DLS non-linear cumulant analysis ---------- 14

Summary of size and zeta potential from DLS ---------- 16

Transmission electron microscopy (TEM) images ---------- 17

Proteolytic stability of stapled P9-PEG conjugates in 1X PBS ---------- 20

AlamarBlue Assay - star-shaped and comb-like conjugates ---------- 29

AlamarBlue Assay - polymer control and comb-like conjugates at 50 µM peptide equivalent concentration ---------- 31

Hemolysis ---------- 32

Peptide synthesis

Stapled P9 monomer (**Figure S1a**) was designed to have a methacrylamide group at the N-terminus with a hexyl linker and was purified by preparative-scale reverse-phase high-performance liquid chromatography (HPLC). The purity of the peptide was assessed by analytical-scale HPLC (≥ 98%, **Figure S1b**), and the synthesis was confirmed by matrix-assisted laser desorption ionization (MALDI-TOF) (**Figure S1c**).

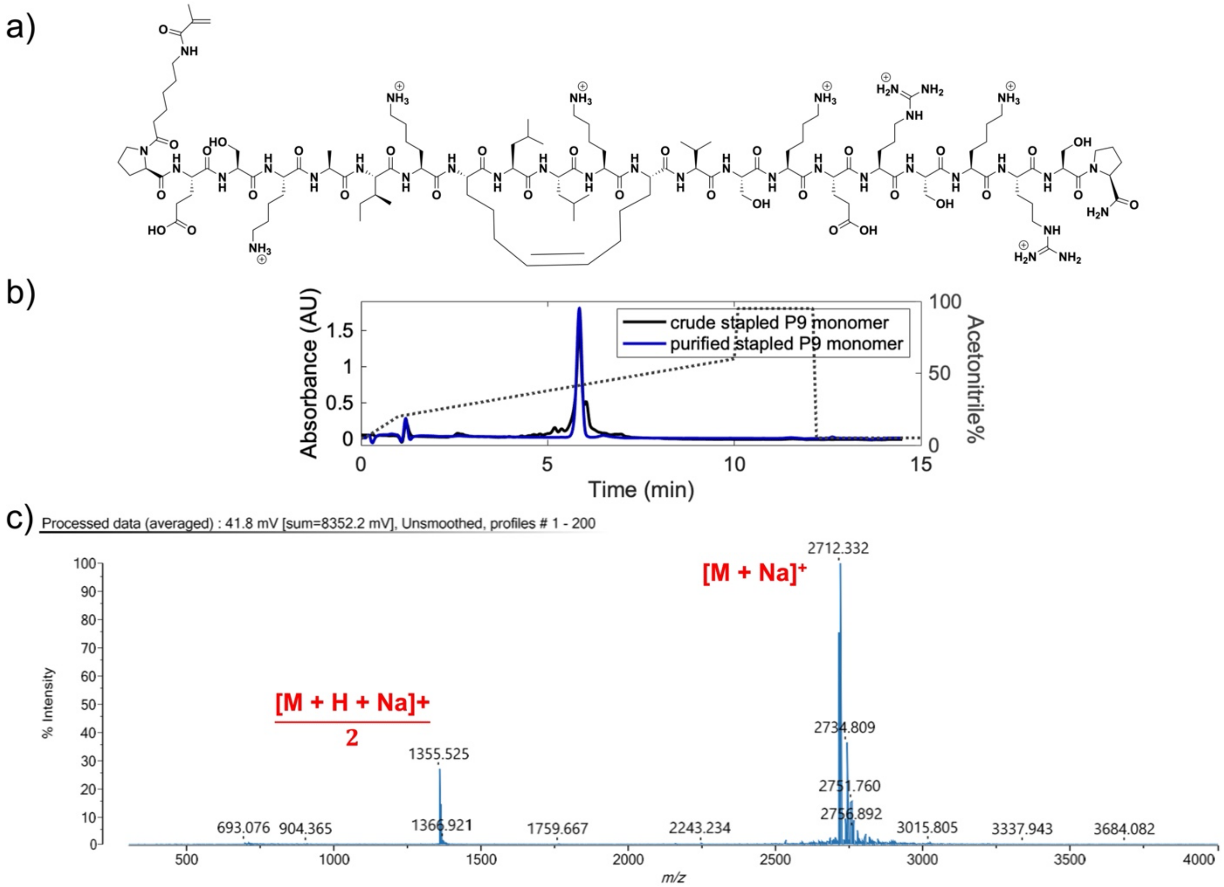

Figure S1. stapled P9 monomer: a) structure; b) analytical HPLC traces of crude and purified stapled P9 monomer; c) MALDI-TOF MS of purified peptide, $[M + Na]^{+}$: calculated 2712.7; found 2712.3.

^{1}H Nuclear magnetic resonance (NMR) spectra of stapled P9 monomer and PEGMA monomers

stapled P9 monomer

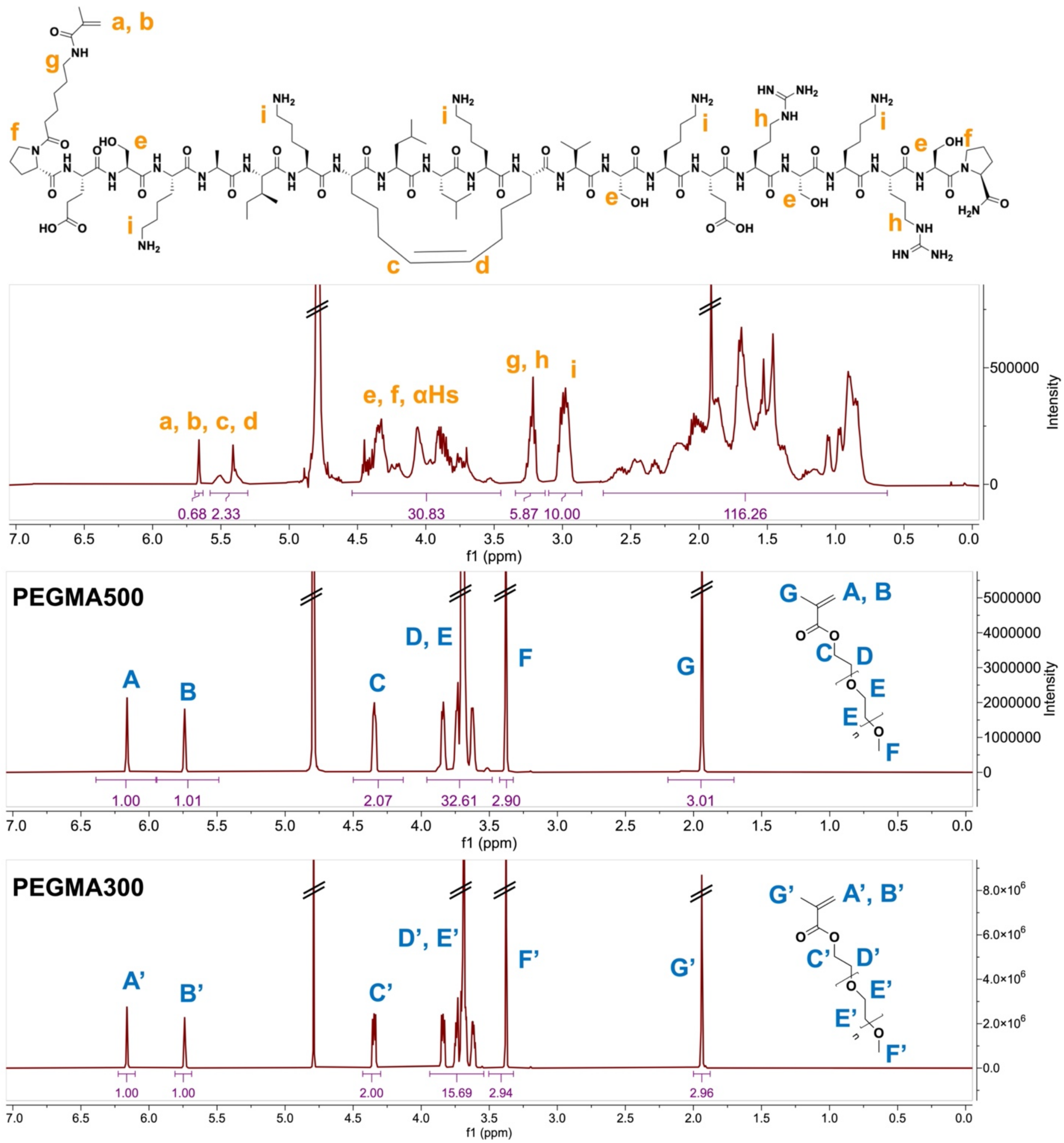

Figure S2. ^{1}H NMR (400 MHz, D_2O) spectra of stapled P9 monomer, PEGMA500, and PEGMA300. The integration of the 5 lysine ε_Hs (3.4 ppm, peak i) was set to 10 (5*2H) in the peptide spectrum. For the PEGMA spectra, the integration of one of the protons on the polymerizable group of PEGMA (6.2 ppm, peak A) was set to 1.

Kinetics of copolymerization of stapled P9 monomer and PEGMA500

We used both size exclusion chromatography (SEC) (**Figure S3a**) and ^{1}H NMR spectroscopy (**Figure S3b**) to monitor the copolymerization of stapled P9 monomer and PEGMA500. SEC traces showed the copolymer dispersity was controlled (Đ<1.3) during copolymerization. A set of representative ^{1}H NMR spectra for kinetics analysis are shown in **Figure S4**. To calculate the conversion of PEGMA, we analyzed the 1H NMR spectra by setting the resonance of the proton on the PEGMA polymerizable group (6.2 ppm) to 1 and compared it to integrations of the methoxy protons at 3.4 ppm at different time points. For the conversion of peptide monomers, we rescale the integration on the NMR spectra by setting one of the protons on the peptide monomer polymerizable group (5.7 ppm) to 1 and compared it to integrations of the Lys εH resonances at 3.0 ppm at different time points. While on PEGMA only the resonance of methoxy protons can be separated from the other peaks from the polymerization mixture for conversion calculation, there are other peaks we can use to determine the conversion of the peptide monomer. We found that conversions calculated from Lys εHs were similar to the values calculated from the peak at 3.22 pm including Arg δHs and the 2 protons on the Ahx spacer next to the methacrylamide (peaks g and h). After subtracting the proton of the polymerizable group on the peptide monomer (peak b, with the assumption that peak a and peak b have similar resonance), we also used the staple protons (peaks c and d) to calculate the conversion and found the results were lower than those the other peaks. While it is possible to have side reactions that would consume the staple protons during polymerization, the low dispersity of the resulting copolymer (**Figure S3a**) suggested the staple protons were not consumed significantly and the lower conversions might be due to those proton resonances not fully integrating on NMR in the buffer-containing mixture. The conversion plot shows that PEGMA polymerized faster than the peptide monomer across the three independent replicates set up independently, suggesting the copolymers have a gradient distribution of the PEG and peptide units.

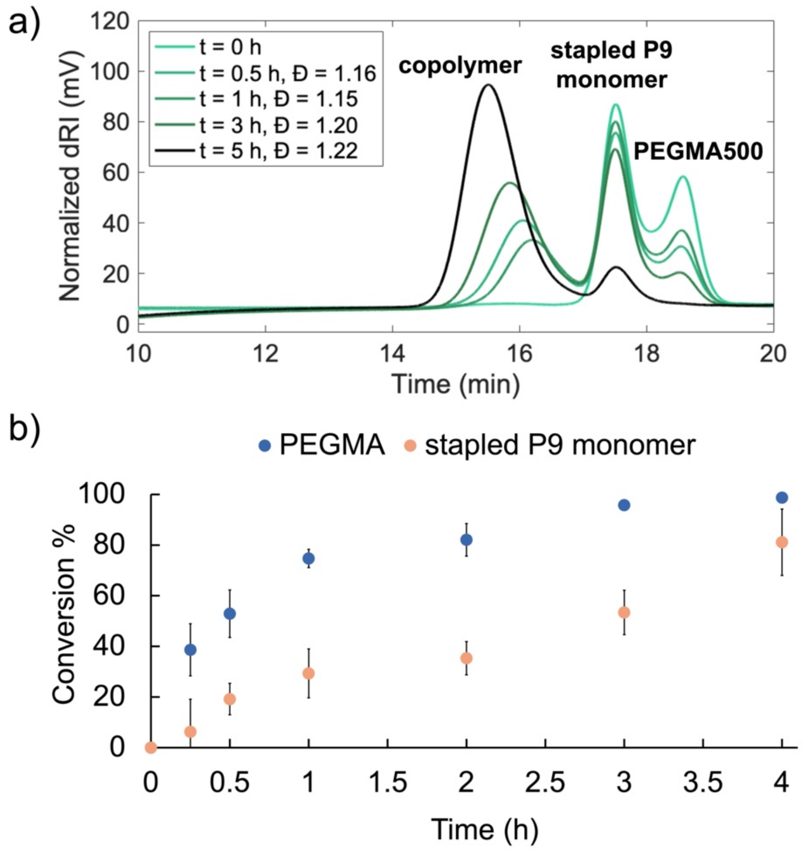

Figure S3. Copolymerization of stapled P9 monomer and PEGMA500: a) SEC traces of the copolymerization mixture over time; b) conversion of both monomers, error bar represents the standard deviation calculated from three replicates from three different polymerization reactions.

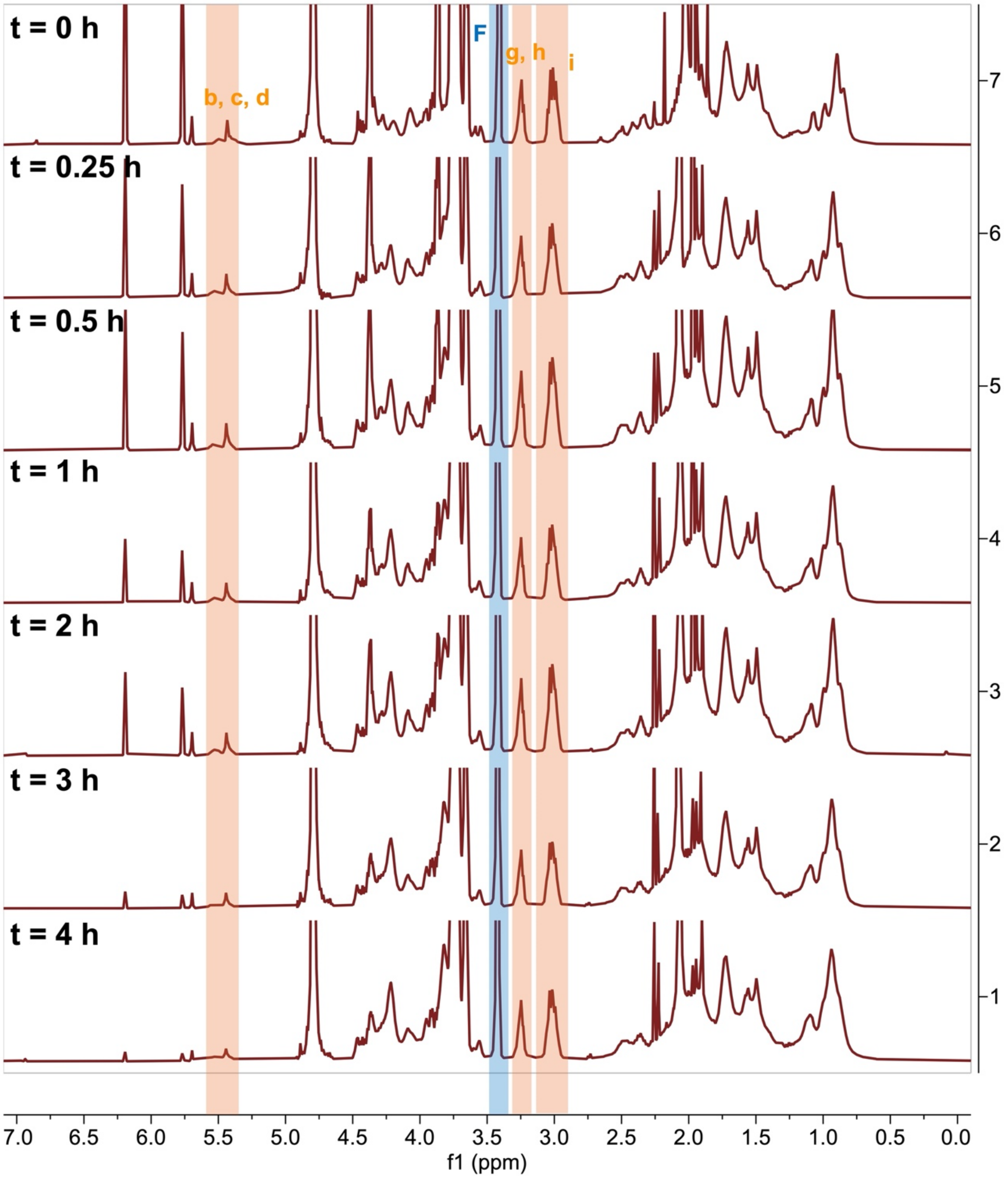

Figure S4. ^{1}H NMR (400 MHz, D_2O) spectra of the copolymerization mixtures. The peaks in the range of 5.3 to 6.3 ppm decrease over time, suggesting the consumption of monomers. We used the methoxy protons at 3.4 ppm (peak F, blue) to calculate the conversion of PEGMA500 and the Lys εHs peak at 3.0 ppm (peak i, orange) to calculate the conversion of stapled P9 monomer. Arg δHs and 2 protons from Ahx (peak g and h) and staple protons (peak c and d) were also used to calculate the conversion of stapled P9 monomer. The peaks are labeled the same as **Figure S2**.

^{1}H NMR spectra of copolymerization mixtures

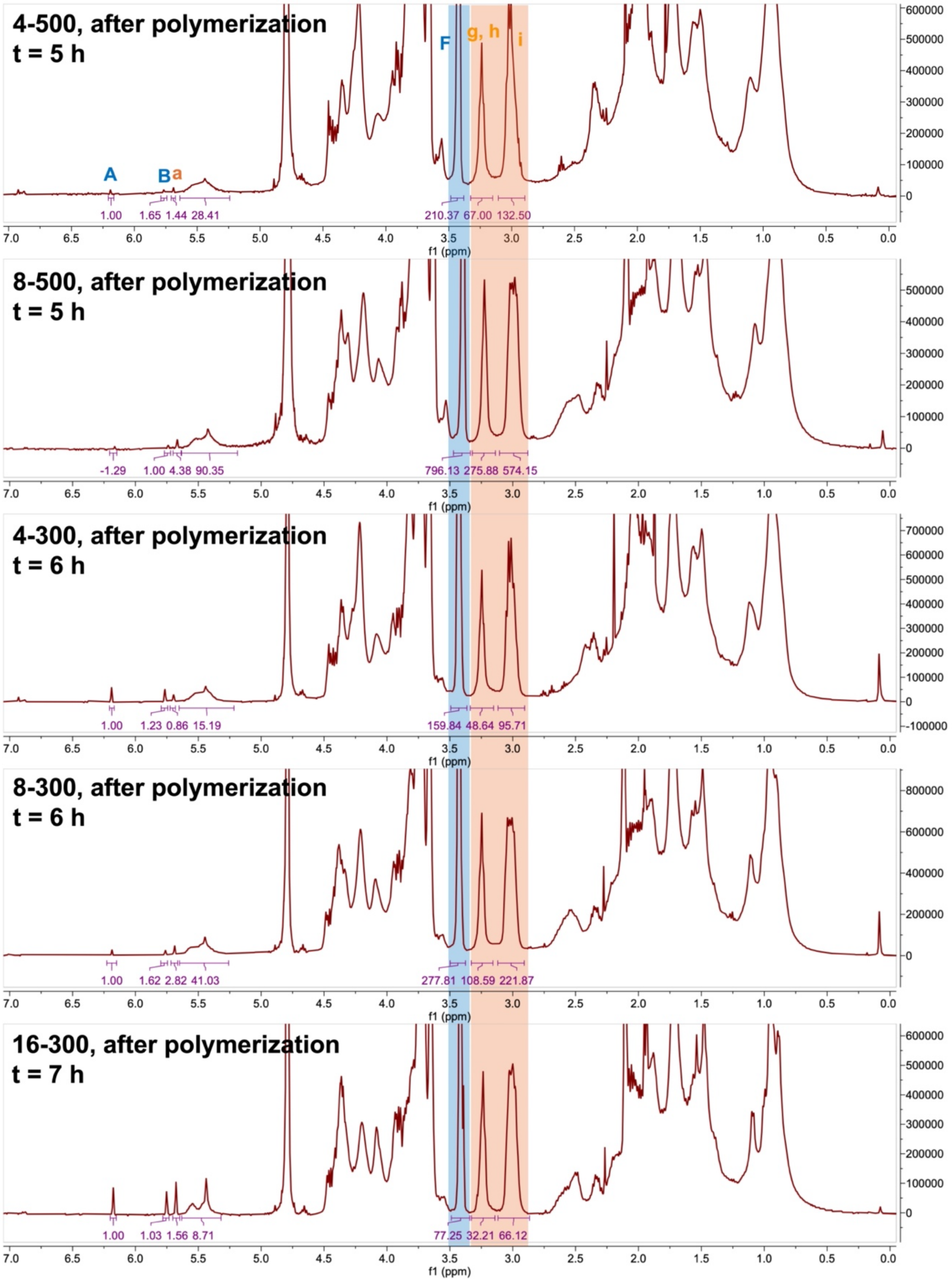

Figure S5. ^{1}H NMR (400 MHz, D_2O) spectra of the copolymerization mixtures. The peaks were labeled the same as Figure **S2**.

^{1}H NMR spectra of purified conjugates

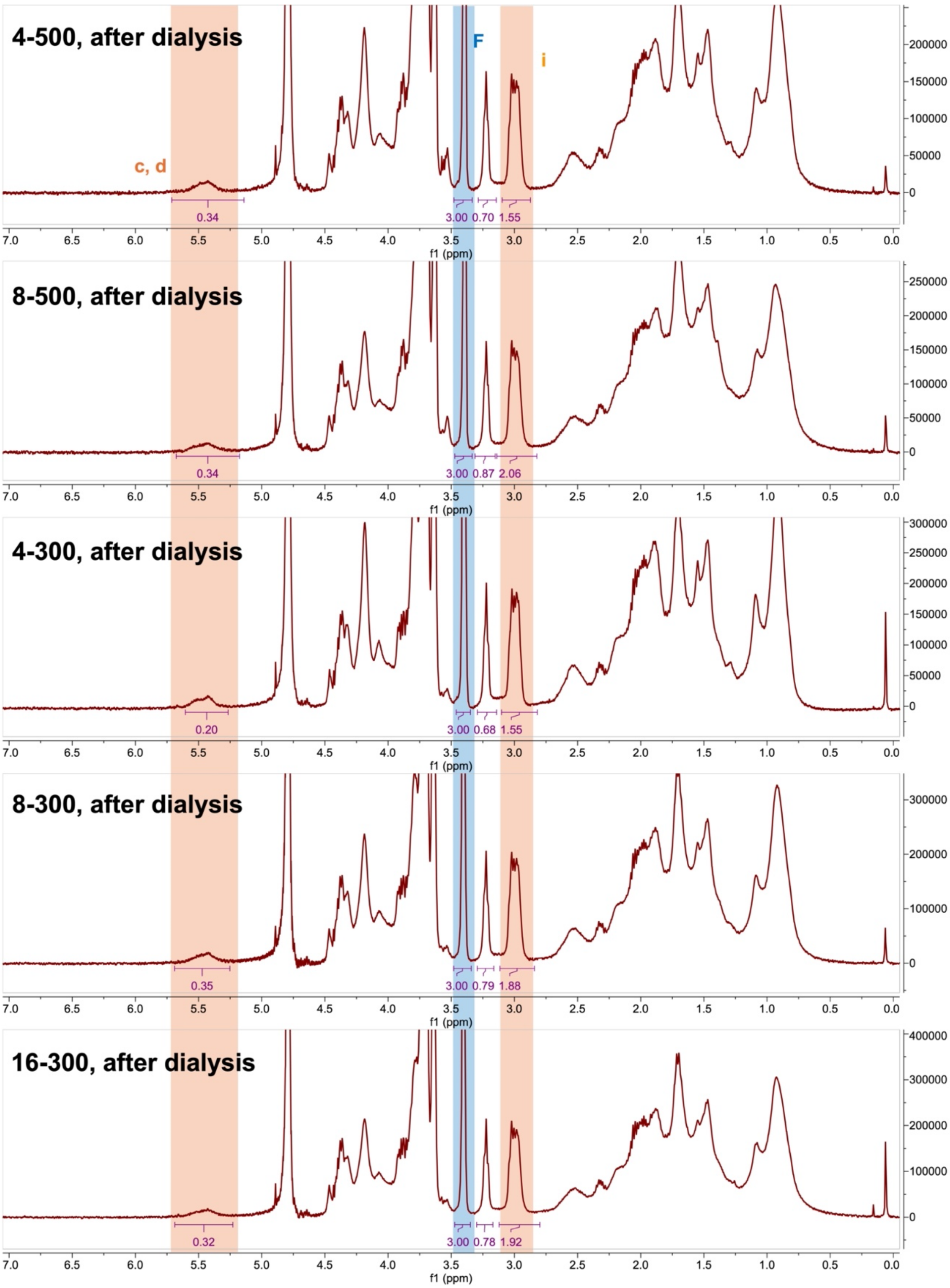

Figure S6. ^{1}H NMR (400 MHz, D_2O) spectra of the comb-like conjugates (5 mg/mL). The peaks are labeled the same as Figure **S2**.

We used the spectra in **Figure S6** to determine the compositions of the purified conjugates after dialysis by comparing the integrations of the methoxy proton resonances on PEG side chains at 3.4 ppm (highlighted in blue, 3 H per repeating unit) to those of the lysine ε_H resonance at 3.0 ppm (highlighted in orange, 10 H per repeating unit). The molar ratios of PEG to peptide monomer (degree of polymerization (DP), DP_{AMP}: DP_{PEGMA}) were consistent with those we calculated based on conversion (**Table S1)**. The ratios of the integration of protons on the staple (2H per repeating unit) to the lysine εHs for most conjugates were close to the expected 0.2:1, indicating that the alkene staples were not consumed during radical copolymerization. Conjugate 4-300 showed a lower fraction of the staple protons, potentially due to the staple protons not fully integrating in this case, as the conjugate maintained a low dispersity that would not be expected if the staples were consumed.

Table S1. Composition of the conjugates calculated from ^{1}H NMR spectra in **Figures S5-S6**

	Int. (PEG)	Int. (Lys)	Int. (staple)	DP_{AMP}: DP_{PEG}	DP_{AMP}: DP_{PEG} (conversion)	Int.(staple): Int.(Lys)
**4-500**	3	1.55	0.34	0.16 : 1	0.16 : 1	0.2 : 1
**8-500**	3	2.06	0.34	0.21 : 1	0.22 : 1	0.17 : 1
**4-300**	3	1.55	0.20	0.16 : 1	0.17 : 1	0.13 : 1
**8-300**	3	1.88	0.35	0.19 : 1	0.20 : 1	0.19 : 1
**16-300**	3	1.92	0.32	0.19 : 1	0.20 : 1	0.17 : 1

Notes: DP_{AMP}:DP_{PEG} was calculated as the ratio of [Int. (Lys)/10]:[Int. (PEG)/3]. DP_{AMP}:DP_{PEGMA} (conversion) was calculated as [target DP_{AMP} x conversion of AMP]:[target DP_{PEGMA} x conversion of PEG].

Synthesis and characterization of conjugate 16-300 analogous polymer control

To ensure that the polymer is not contributing to the antimicrobial activity, we synthesized a comb homopolymer containing only the PEGMA monomer and evaluated its antimicrobial activity using the alamarBlue assay. We opted for a homopolymer analogous to the 16-300 conjugate since this conjugate was the most active against bacteria. The homopolymer was synthesized using RAFT polymerization with the same target DP of PEGMA monomer as the 16-300 conjugate. The amounts of reagents used are listed in **Table S2**.

Table S2. Reagent amounts used for the RAFT polymerization of the polymer control.

	equiv.	conc./ mass (mM/ mg/mL)
**PEGMA**	80	285.7 (85.7)
**CTA**	1	3.6 (1.1)
**Initiator (LPTP)**	0.3	1.1 (0.3)

The reaction was conducted using the same method described for the synthesis of the comb conjugates in 5 mL of acetate buffer (pH = 5) as the solvent. The polymerization was performed for 6 h, and the reaction mixture was lyophilized for further analysis. To calculate conversion, we used ^{1}H NMR spectroscopy. The ^{1}H NMR spectra of the polymer showed the disappearance of the peaks associated with the polymerizable methacrylamide at 6.2 ppm and 5.8 ppm, indicating 100% conversion of the PEGMA monomer (**Figure S7**). Given the full conversion of the PEGMA, we assumed the calculated DP of the polymer to be the same as the target DP (DP_{target} = [M]: [CTA]). The remaining polymer solution was redissolved in water and dialyzed for 2 days with constant water changes to remove impurities for future activity studies.

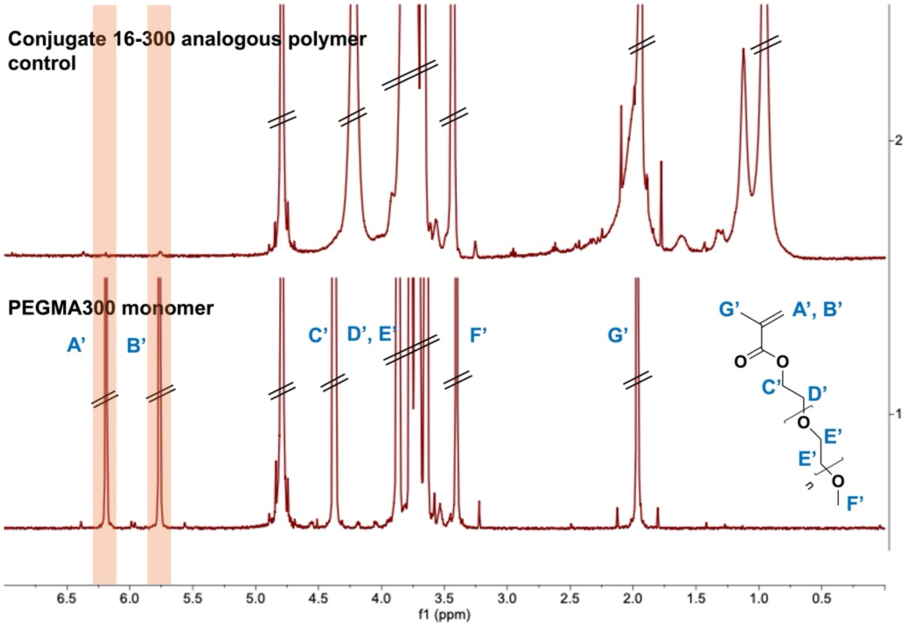

Figure S7. ^{1}H NMR (400 MHz, D_2O) spectra of the (top) polymer control, and (bottom) PEGMA300 monomer. The highlighted peaks at 6.20 and 5.80 ppm correspond to the polymerizable groups on the monomer. The peaks are labeled the same as Figure **S2**.

The SEC trace of the resulting polymer is shown below (**Figure S8**). Relative to PMMA standards, we obtained M_n = 25400 Da and Đ = 1.57. We note that the dispersity value is slightly higher than 1.3 (the highest dispersity typically accepted for controlled polymerization) and attribute this to the low [CTA] to [LPTP] ratio used for the synthesis of conjugates.

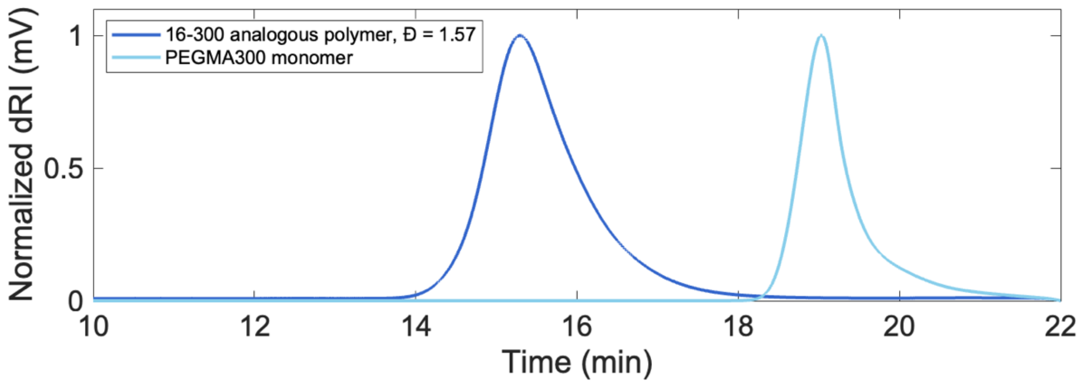

Figure S8. The SEC trace of the polymer control (dark blue) and the PEGMA300 monomer (light blue).

Circular dichroism (CD) spectra of stapled P9, stapled P9 monomer, and the comb-like conjugates

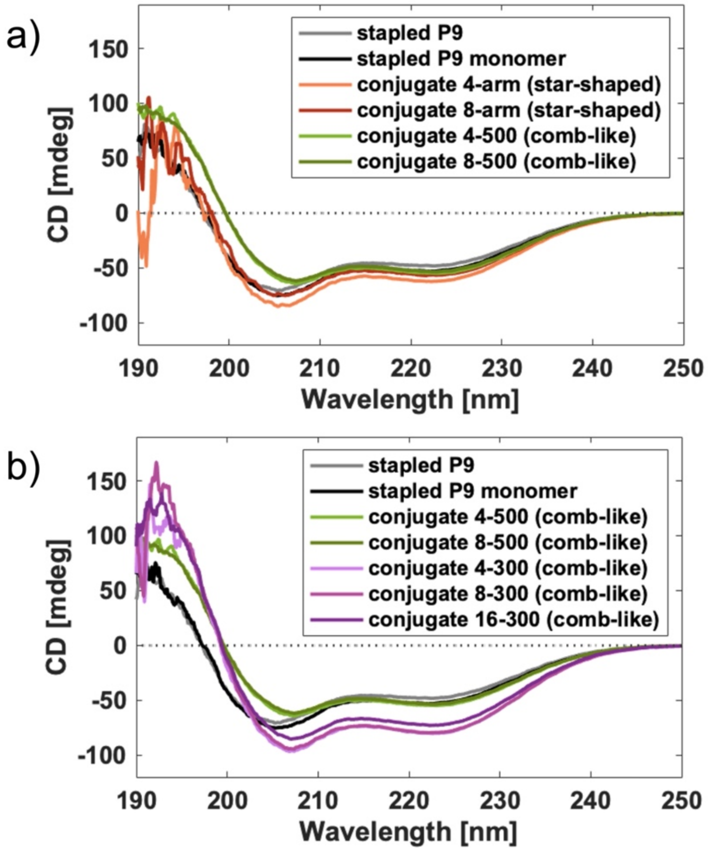

Figure S9. CD spectra of stapled P9, stapled P9 monomer, and the conjugates at 200 μM peptide equivalent concentration in 10 mM PBS: a) comparison among comb-like conjugates and b) comparison across star-shaped and comb-like architectures.

Dynamic light scattering (DLS) results of stapled P9 and the comb-like conjugates

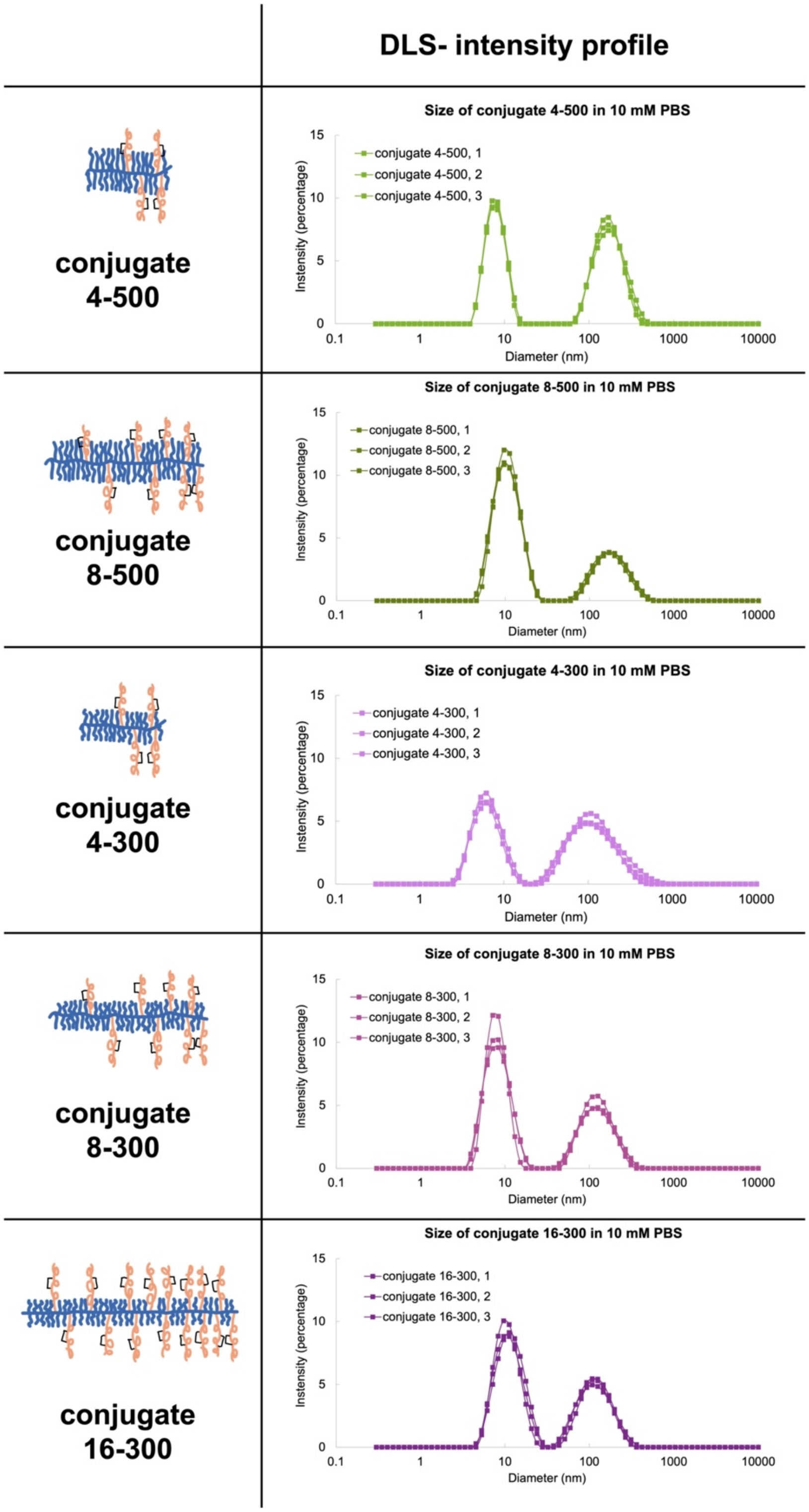

Figure S10. DLS intensity profiles of conjugates at 200 µM peptide equivalent in 10mM PBS.

DLS non-linear cumulant analysis

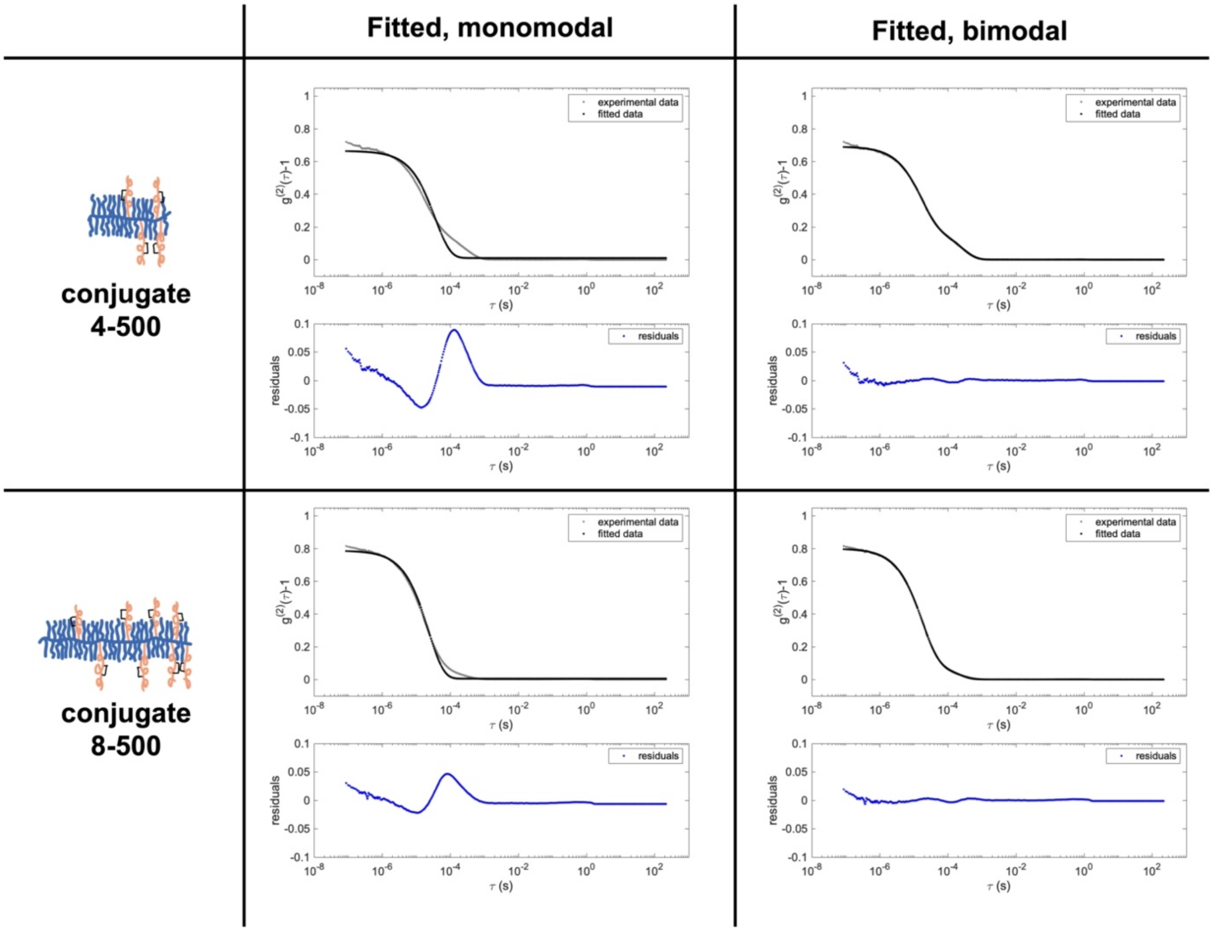

Figure S11. Fitted correlograms and residuals (left: monomodal; right: bimodal) generated by comparing the experimental measurements to the fitted data of conjugates 4-500 and 8-500.

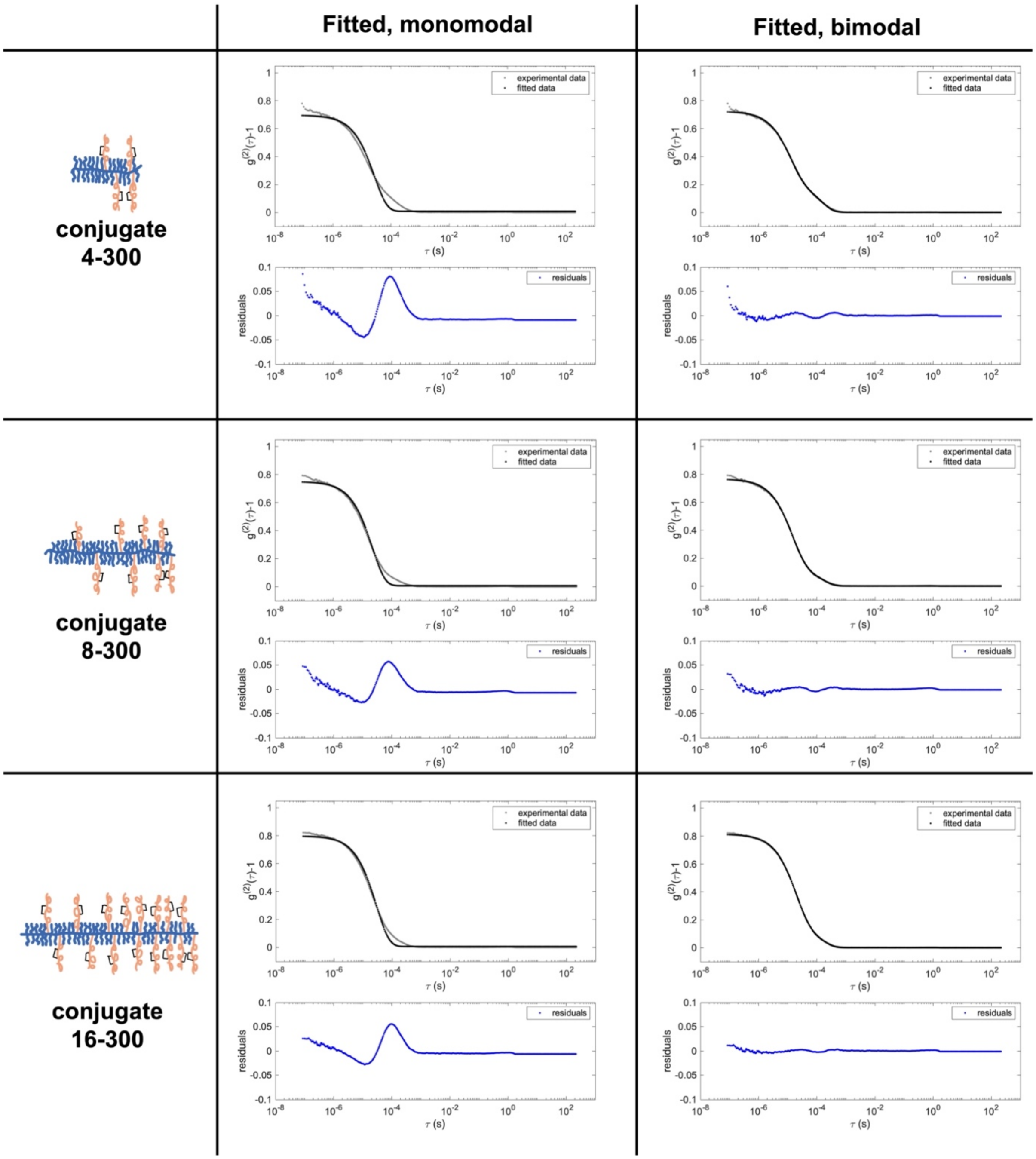

Figure S12. Fitted correlograms and residuals (left: monomodal; right: bimodal) generated by comparing the experimental measurements to the fitted data of conjugates 4-300, 8-300, and 16-300.

Summary of size and zeta potential from DLS

Table S3. Zeta potential and size (diameter) of stapled P9 and conjugates.

	stapled P9	conjugate 4-500	conjugate 8-500	conjugate 4-300	conjugate 8-300	conjugate 16-300
**Mass conc. (mg/mL)**	0.67	1.33	1.16	1.05	1.00	1.00
**Zeta potential (mV)**	27 ± 6.4	21 ± 1.8	22 ± 0.9	26 ± 2.1	21 ± 2.6	30 ± 2.3
**Count rate, DLS (kcps)**	-	1134 ± 25	1146 ± 5	588± 43	721 ± 7	1378 ± 6
**Diameter, DLS z-average (nm)**	-	16.6 ± 0.1	13.8 ± 0.2	13.9 ± 0.6	12.5 ± 0.2	17.7 ± 0.4
**Diameter, fitted, monomodal (nm)**	-	24.7	15.9	18.2	14.9	19.8
**Diameter, DLS intensity 1 (nm)**	-	8.1 ± 0.1	11.0 ± 0.1	6.6 ± 0.2	8.4 ± 0.2	11.6 ± 0.6
**Diameter, DLS intensity 2 (nm)**	-	180.5 ± 7.1	191.6 ± 9.8	133.3 ± 12.2	132.5 ± 4.4	126 ± 3.6
**Diameter, fitted, bimodal 1 (nm)**	-	7.2	8.9	5.4	6.9	9.1
**Diameter, fitted, bimodal 2 (nm)**	-	162	120.2	92.2	92.9	92.5

Measurements were taken at a peptide equivalent concentration of 200 μM in 10 mM PBS in triplicate. Z-average values and peak intensity averages are reported from DLS analysis (**Figure S10**). The correlograms were fitted by two models to calculate the diameter populations (**Figure S11-12**).

Transmission electron microscopy (TEM) images

To prepare the TEM grids, we first added 3 µL of the conjugate sample (200 µM peptide equivalent concentrations in 10 mM PBS, pH = 7.4) to the grid and washed times with one drop of water (10 µL) per wash, however, it is difficult for the grids to retain clear structures that can be observed by TEM (Figure S13). Considering the conjugates may be washed away during sample preparation, we then reduced the time of water washing to once to retain the materials. The representative TEM images are shown in Figures S14-15.

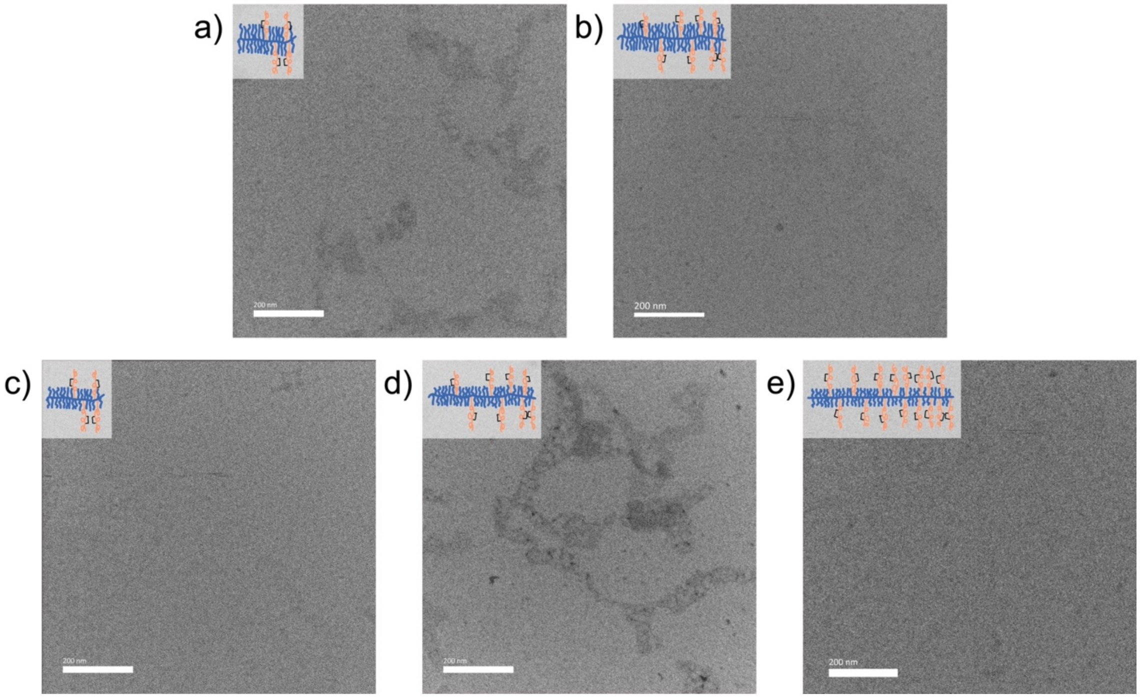

Figure S13. Representative TEM images of a) conjugate 4-500; b) conjugate 8-500; c) conjugate 4-300; d) conjugate 8-300; and e) conjugate 16-300. Grids were prepared with three water washes. Scale bar: 200 nm.

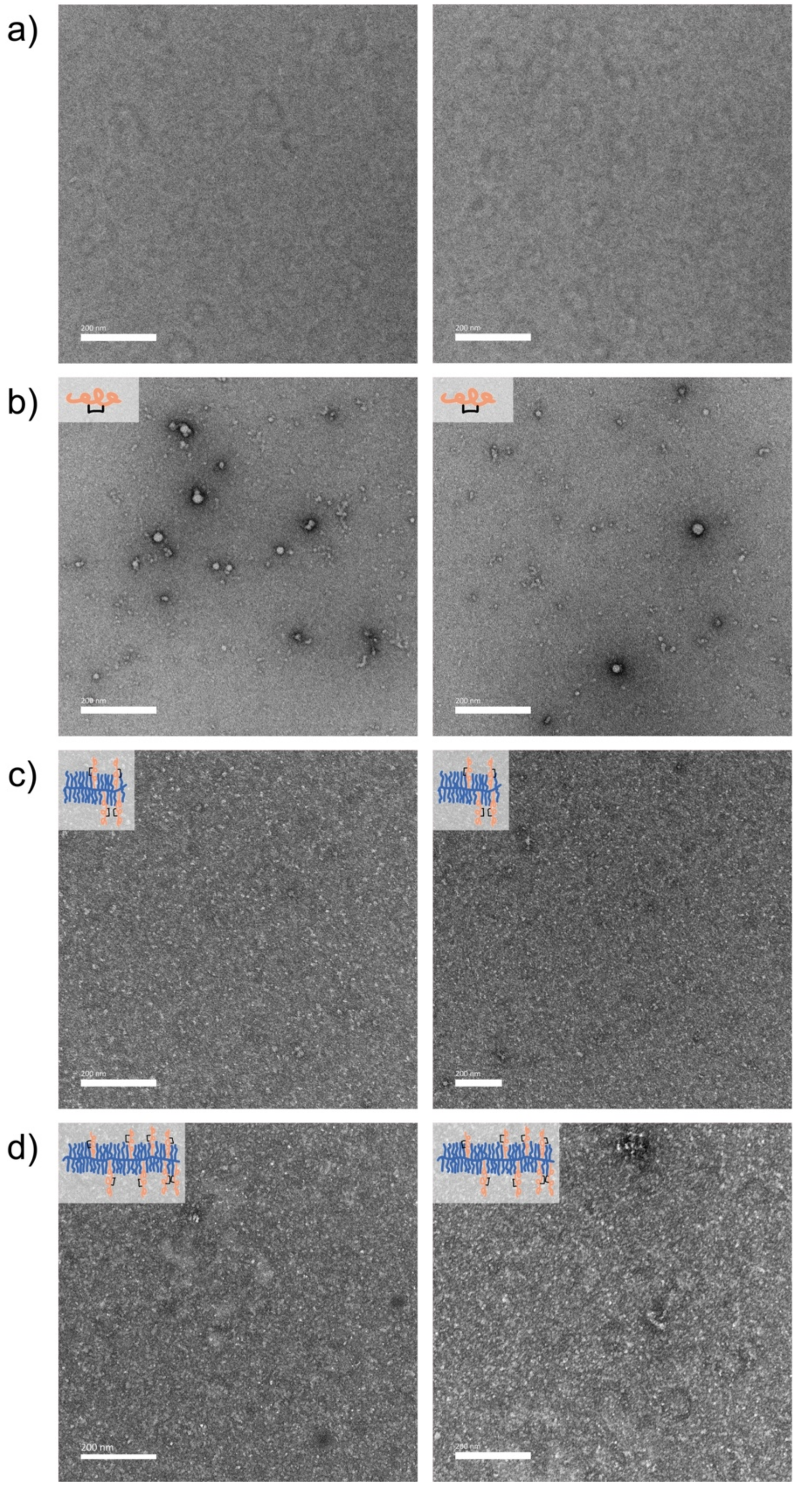

Figure S14. Representative TEM images of a) buffer alone; b) stapled P9 monomer; c) conjugate 4-500; and d) conjugate 8-500. Grids were prepared and washed once with water. Two independent fields are shown for each condition. Scale bar: 200 nm.

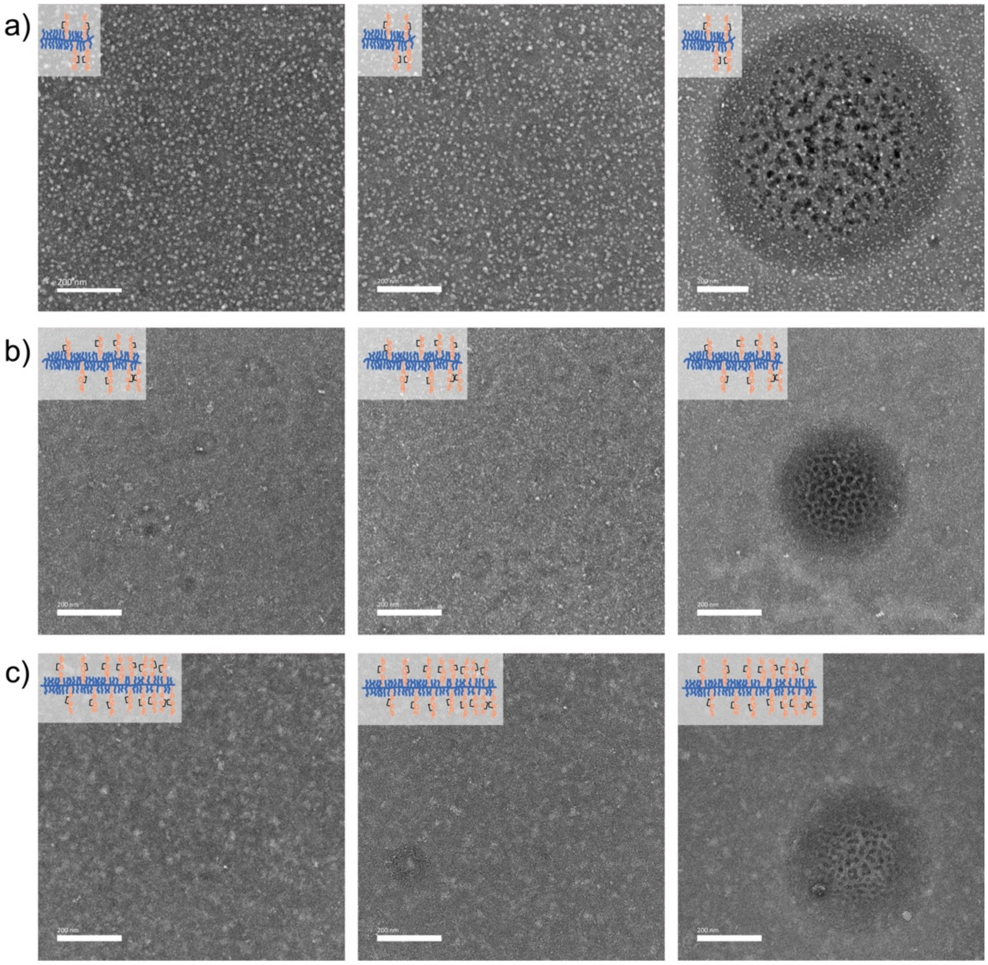

Figure S15. Representative TEM images of a) conjugate 4-300; b) conjugate 8-300; and c) conjugate 16-300. Grids were washed three times with water. Scale bar: 200 nm.

The TEM images of the polymerization mixture for preparing conjugate 4-500 are shown in **Figure S16.**

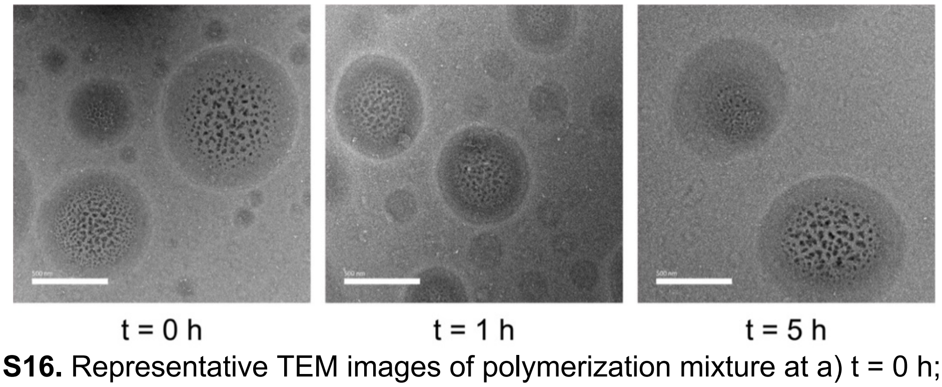

Figure S16. Representative TEM images of polymerization mixture at a) t = 0 h; b) t = 1 h; and c) t = 5 h. Grids were prepared and washed three times with water. Scale bar: 200 nm.

Proteolytic stability of stapled P9-PEG conjugates in 1X PBS

The HPLC traces of Proteinase K treated stapled P9 are provided in **Figure S17**.

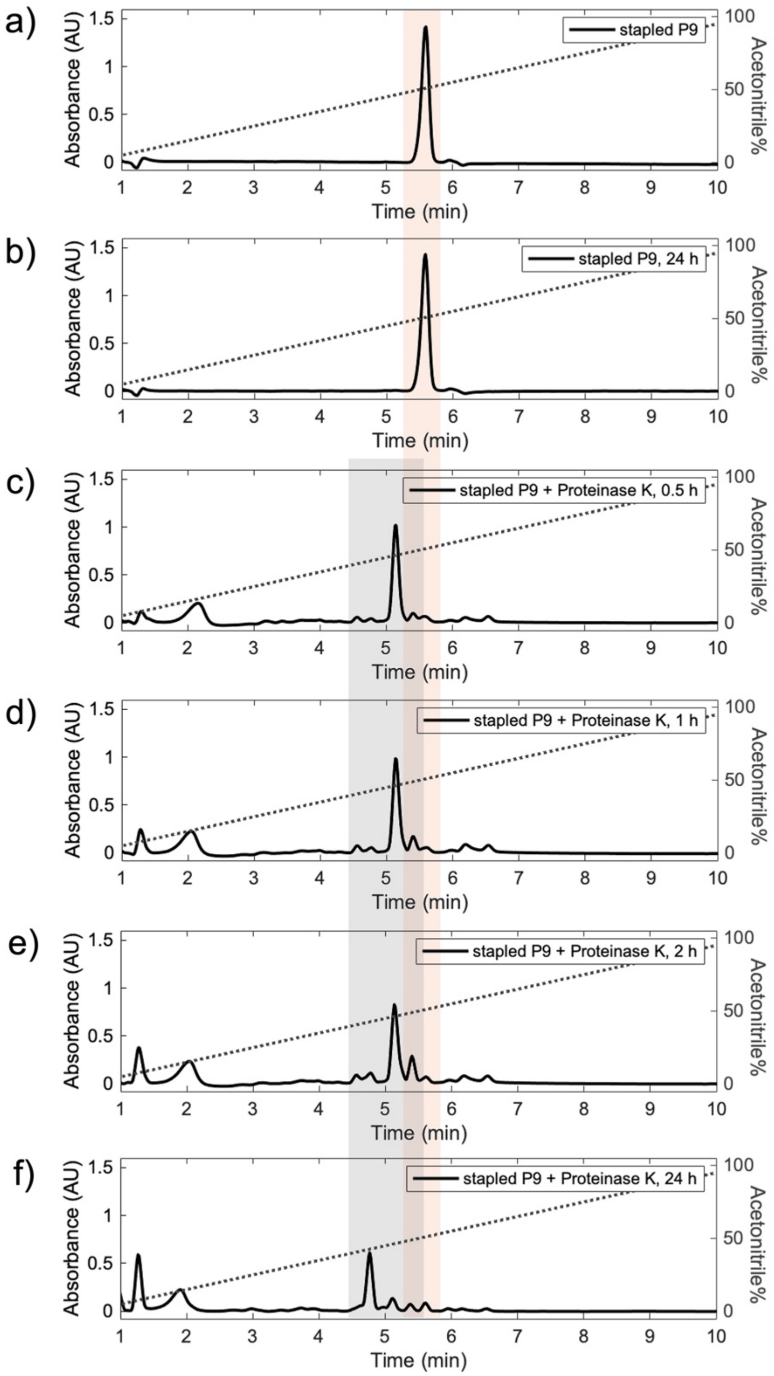

Figure S17. HPLC traces of stapled P9: a) alone in 1X PBS, b) alone in 1X PBS after 24 h, or c-f) incubated with Proteinase K for 0.5, 1, 2, or 24 h in 1X PBS. The area highlighted in orange shows the intact peptide and the area highlighted in grey was used for integration.

The HPLC traces of Proteinase K treated conjugate 4-500 are provided in **Figure S18**.

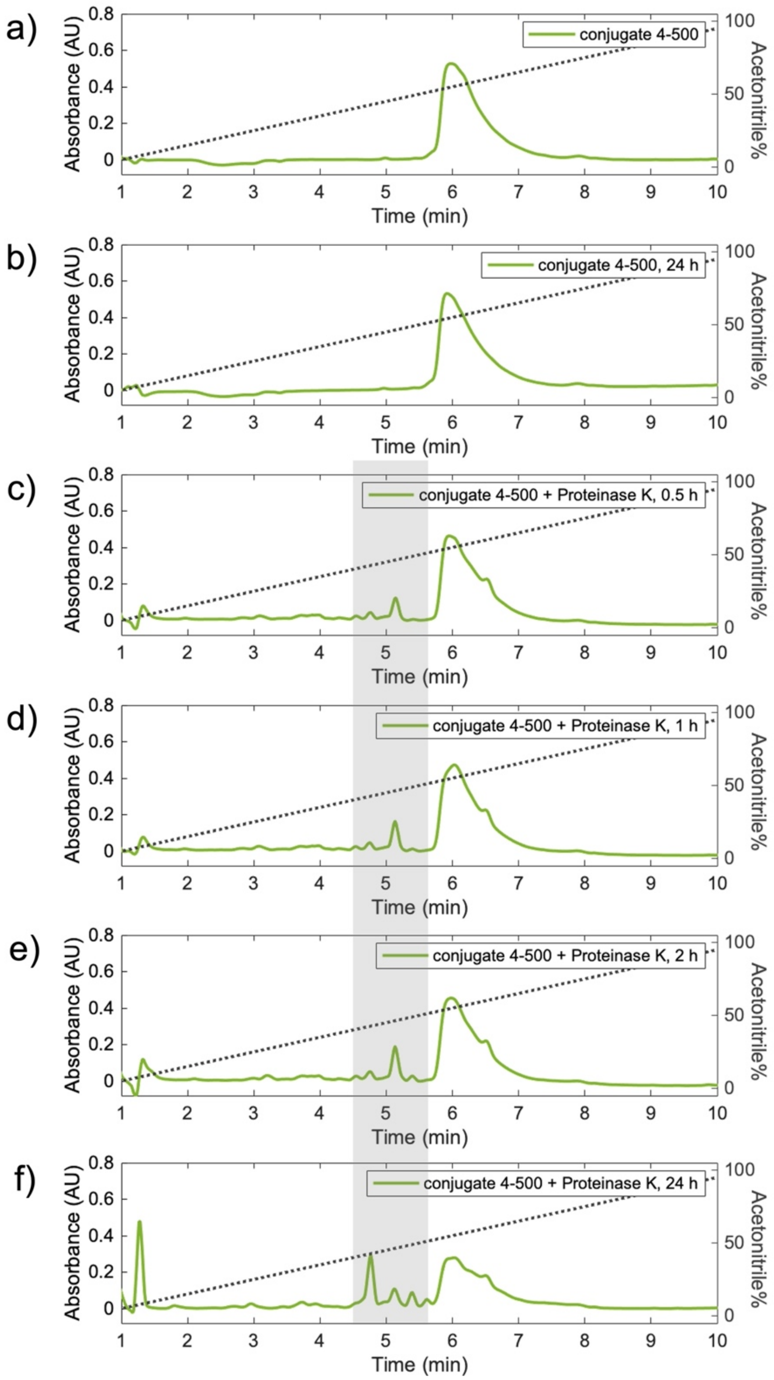

Figure S18. HPLC traces of conjugate 4-500: a) alone in 1X PBS, b) alone in 1X PBS after 24 h, or c-f) incubated with Proteinase K for 0.5, 1, 2, or 24 h in 1X PBS. The area highlighted in grey was used for integration.

The HPLC traces of Proteinase K treated conjugate 8-500 are provided in **Figure S19.**

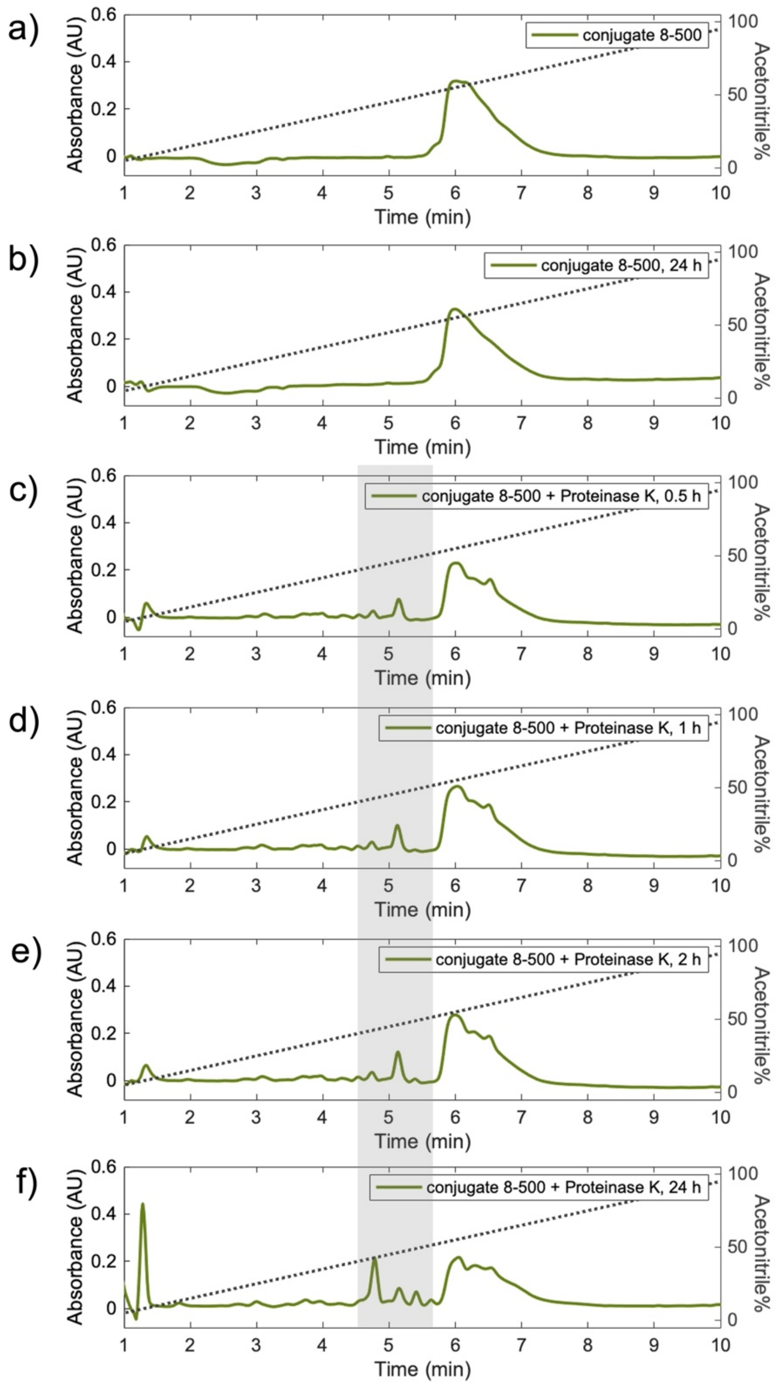

Figure S19. HPLC traces of conjugate 8-500: a) alone in 1X PBS, b) alone in 1X PBS after 24 h, or c-f) incubated with Proteinase K for 0.5, 1, 2, or 24 h in 1X PBS. The area highlighted in grey was used for integration.

The HPLC traces of Proteinase K treated conjugate 4-300 are provided in **Figure S20**.

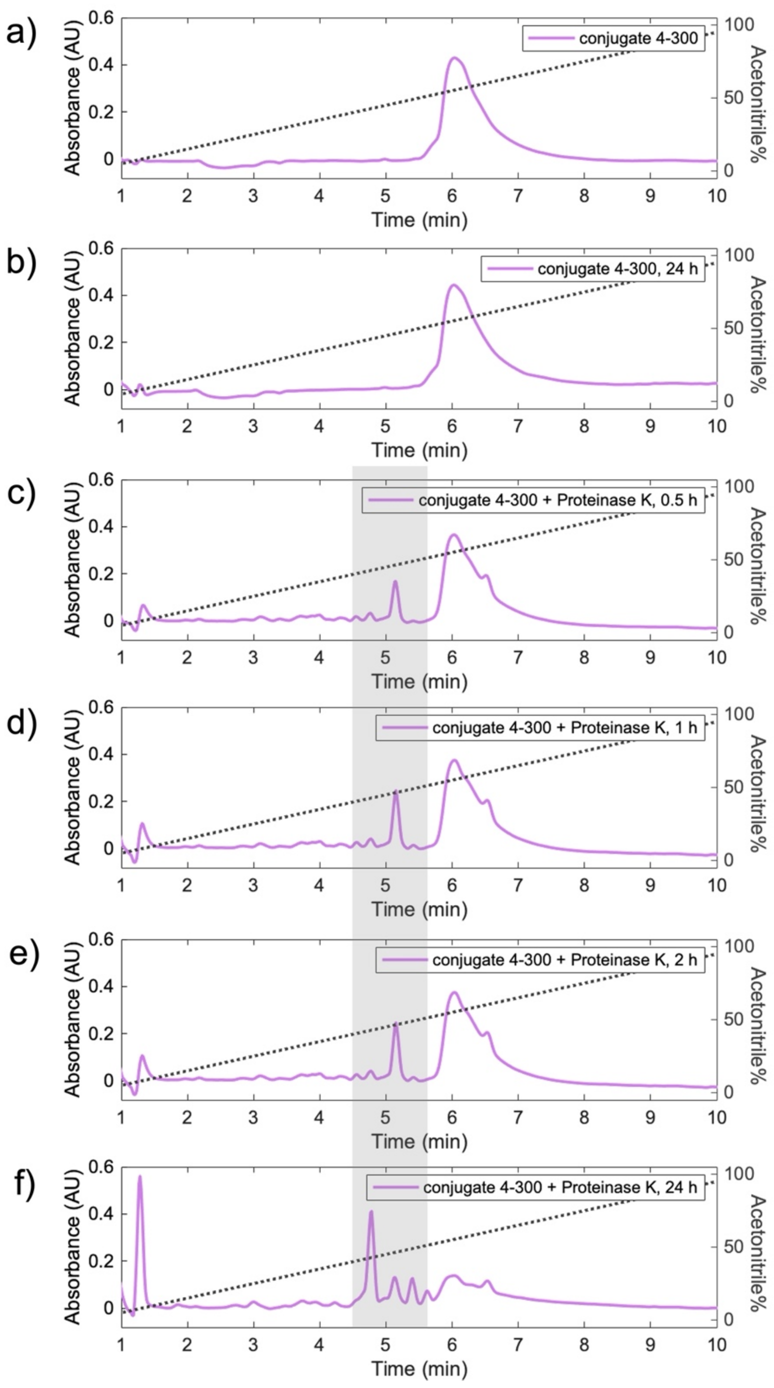

Figure S20. HPLC traces of conjugate 4-300: a) alone in 1X PBS, b) alone in 1X PBS after 24 h, or c-f) incubated with Proteinase K for 0.5, 1, 2, or 24 h in 1X PBS. The area highlighted in grey was used for integration.

The HPLC traces of Proteinase K treated conjugate 8-300 are provided in **Figure S21.**

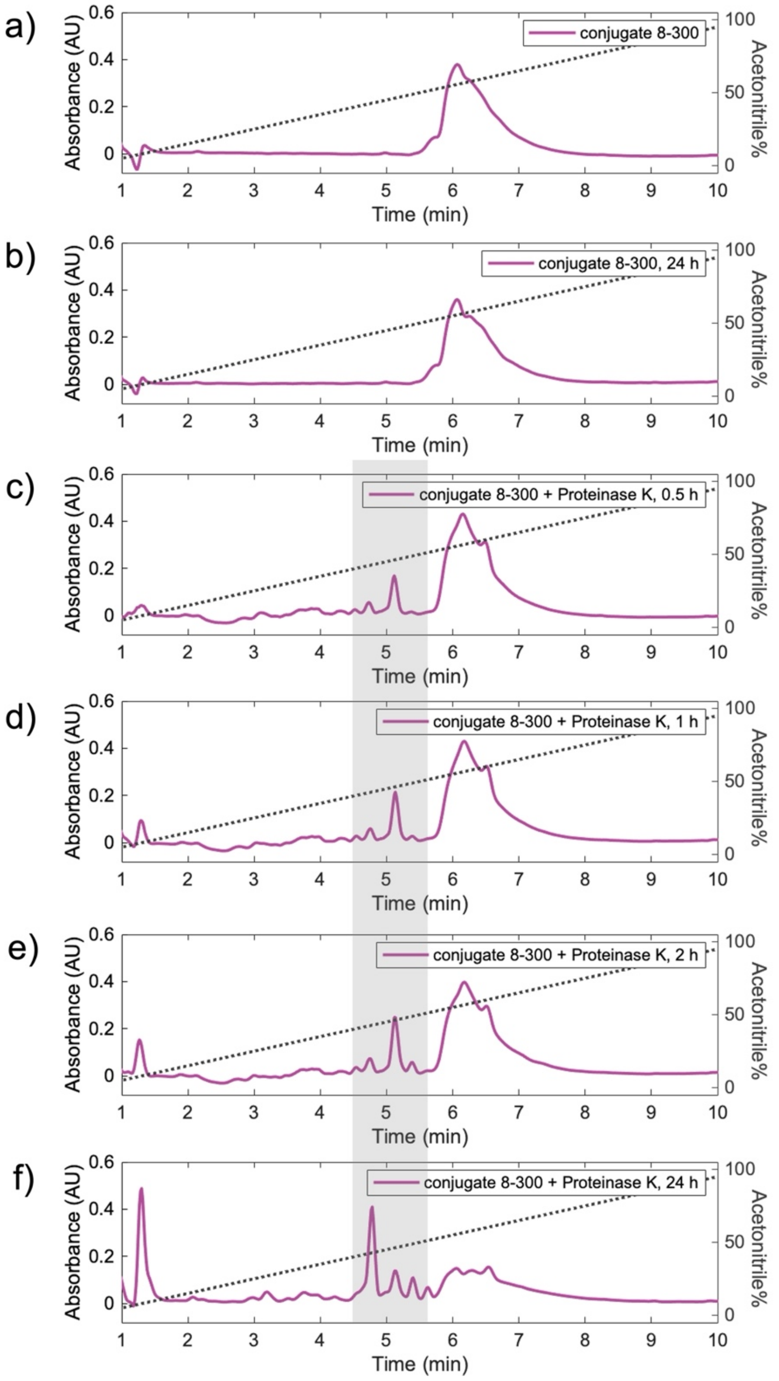

Figure S21. HPLC traces of conjugate 8-300: a) alone in 1X PBS, b) alone in 1X PBS after 24 h, or c-f) incubated with Proteinase K for 0.5, 1, 2, or 24 h in 1X PBS. The area highlighted in grey was used for integration.

The HPLC traces of Proteinase K treated conjugate 16-300 are provided in **Figure S22.**

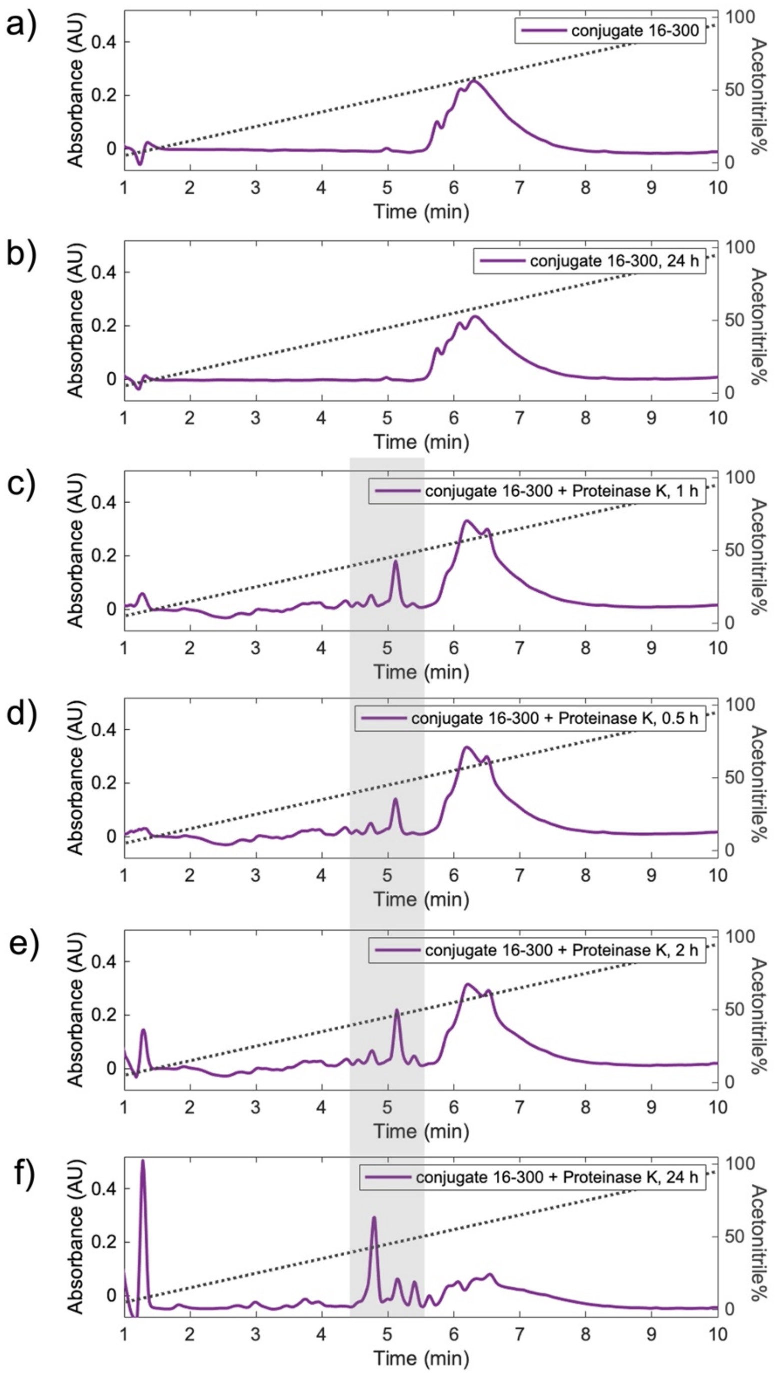

Figure S22. HPLC traces of conjugate 16-300: a) alone in 1X PBS, b) alone in 1X PBS after 24 h, or c-f) incubated with Proteinase K for 0.5, 1, 2, or 24 h in 1X PBS. The area highlighted in grey was used for integration.

The HPLC traces of Proteinase K treated conjugate 4-arm are provided in **Figure S23.**

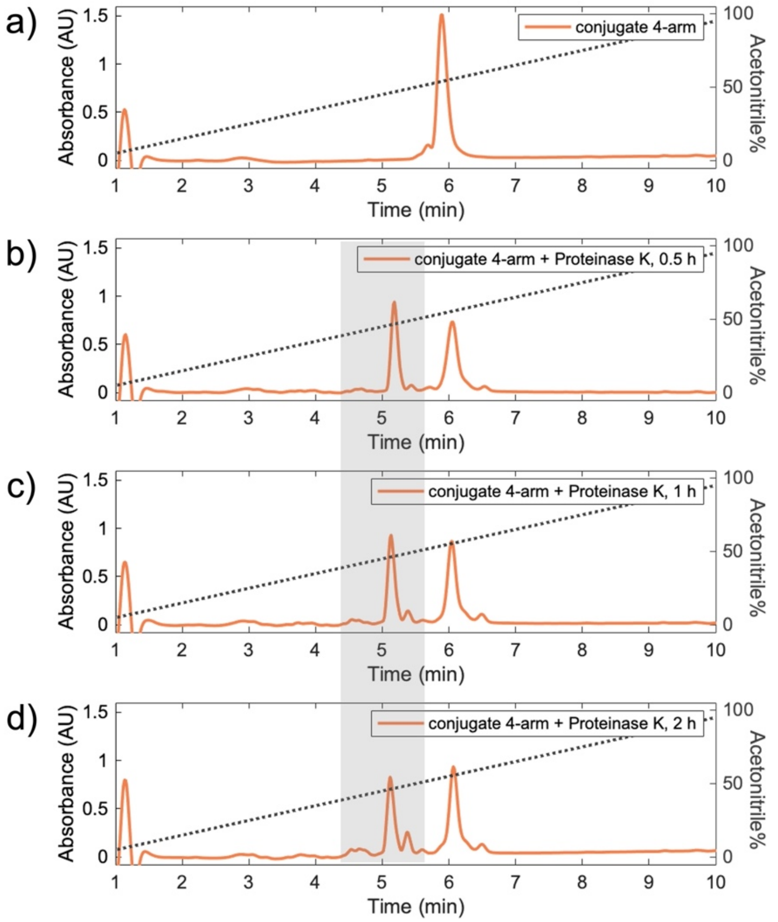

Figure S23. HPLC traces of conjugate 4-arm: a) alone in 1X PBS or b-d) incubated with Proteinase K for 0.5, 1, or 2 h in 1X PBS. The area highlighted in grey was used for integration.

The HPLC traces of Proteinase K treated conjugate 8-arm are provided in **Figure S24.**

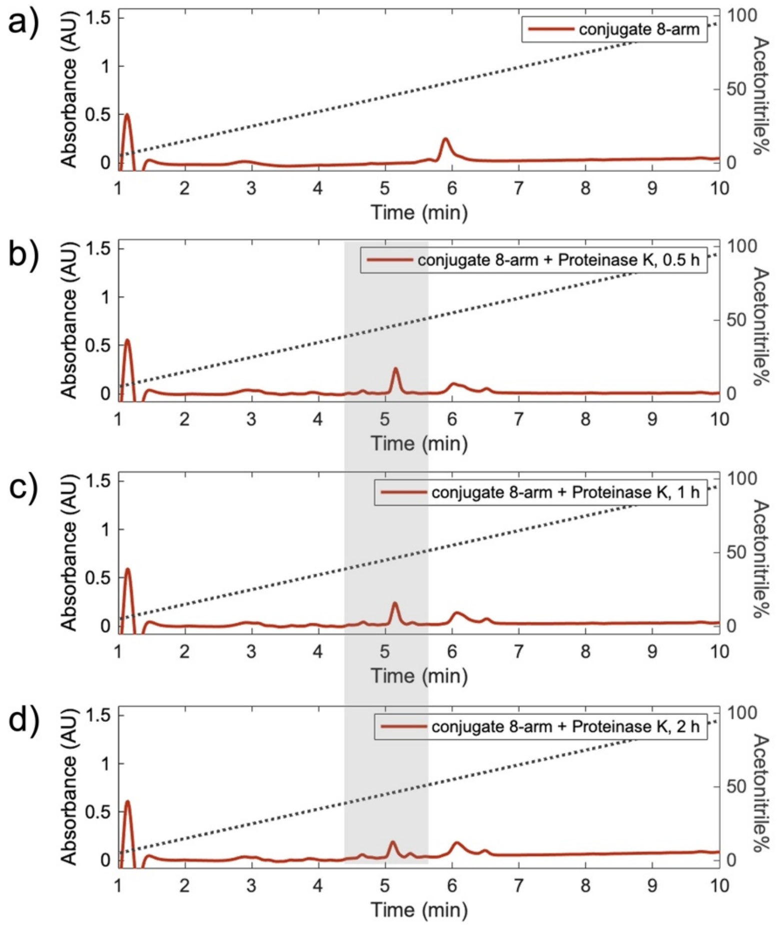

Figure S24. HPLC traces of conjugate 8-arm: a) alone in 1X PBS or b-d) incubated with Proteinase K for 0.5, 1, or 2 h in 1X PBS. The area highlighted in grey was used for integration.

The integrations of peaks calculated by Empower HPLC software are listed in **Tables S4-5.**

Table S4. Summary of raw HPLC peak area data (absorbance x time, A.U. x min) of the peptide fragments released from peptide/conjugate treated with Proteinase K in 1X PBS after 0.5, 1, 2, and 24 h.

	stapled P9	conjugate 4-500	conjugate 8-500	conjugate 4-300	conjugate 8-300	conjugate 16-300	conjugate 4-arm	conjugate 8-arm
**t = 0 h**	0	0	0	0	0	0	0	0
**t = 0.5 h**	7193468	1256251	869174	1512785	1573941	1230921	7040040	1691736
**t = 1 h**	7304566	1594031	1002559	2072610	2121330	1970332	7250309	2207030
**t = 2 h**	8368090	1865852	1422504	2896849	2672391	2553346	8291788	1985596
**t = 24 h**	5746567	3319182	2469840	4670705	4665016	4212046	-	-

Table S5. Summary of raw HPLC peak area data (absorbance x time, A.U. x min) of peptide/conjugate treated with Proteinase K in 1X PBS after 0.5, 1, 2, and 24 h.

	stapled P9	conjugate 4-500	conjugate 8-500	conjugate 4-300	conjugate 8-300	conjugate 16-300	conjugate 4-arm	conjugate 8-arm
**control**	11846268	21894577	16684078	20160640	20649211	18051047	17909022	3459391
**t = 0.5 h**	7193468	21084419	13751562	18419728	19500836	16784639	8181820	2161471
**t = 1 h**	7304566	20687636	16198067	18119869	18935319	16833633	9711549	2450182
**t = 2 h**	8368090	20142426	15916688	15929425	17860271	16326938	8052220	2488585
**t = 24 h**	5746567	12912043	12138090	6633897	9586533	9824129	-	-

AlamarBlue Assay - star-shaped and comb-like conjugates

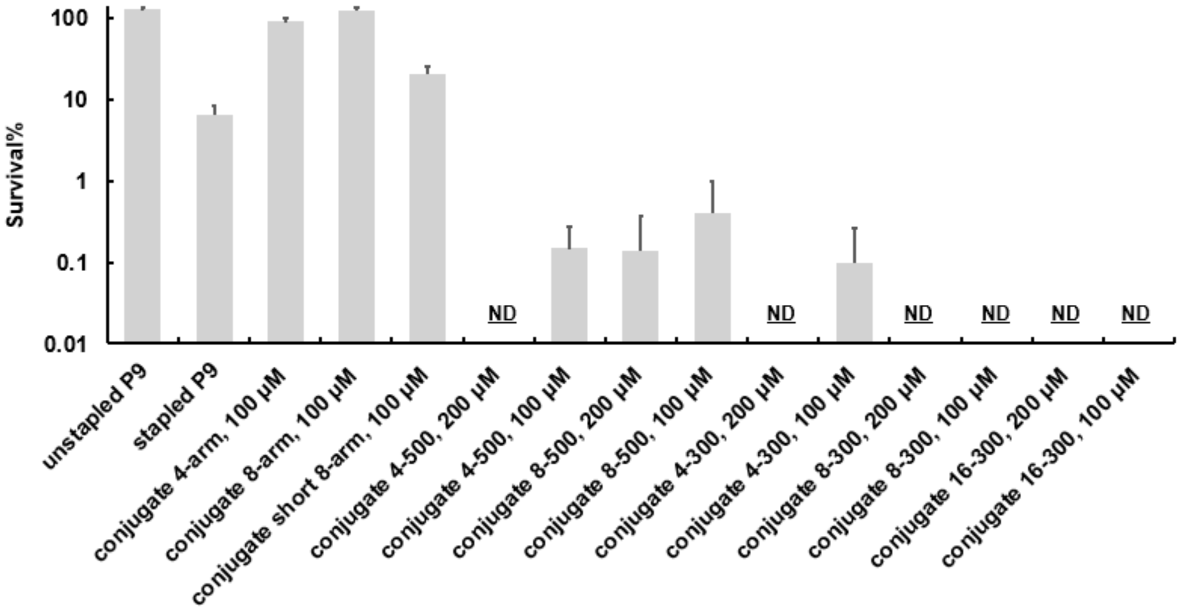

Figure S25. Bacterial survival (%) after treatment with the indicated AMP or conjugate (peptide equivalent concentrations = 200 or 100 µM in RPMI medium). The assays were performed in triplicate. Bars indicate the average percent survival. Error bars represent the standard deviation. ND: none detected (bacterial survival < .01%). Unstapled P9 was chosen as a negative control that did not show antimicrobial activity.

Representative picture of a microplate used for the alamarBlue assay (**Figure S26**). While we did not observe significant differences between the comb-like conjugates at 100 µM peptide, we noticed that after overnight incubation only samples treated with conjugate 16-300 remained blue, suggesting that 16-300 was the most active of the tested conjugates and killed most bacteria after the first 2 h exposure.

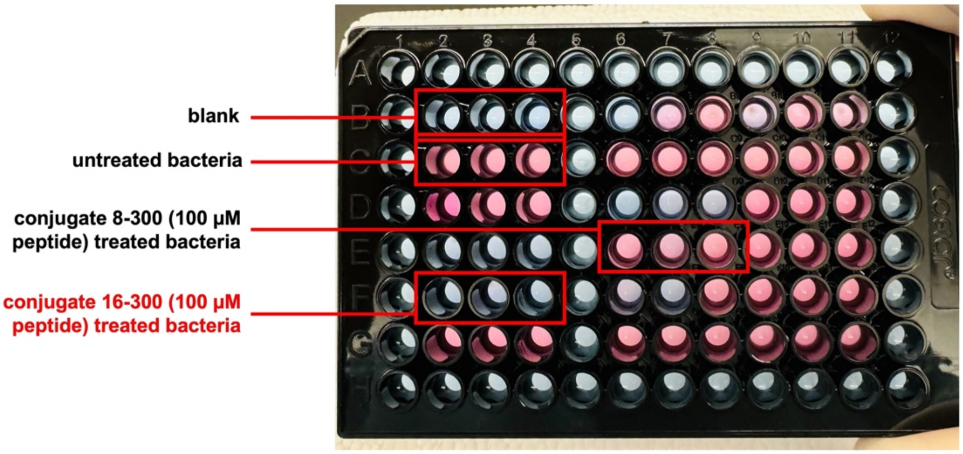

	1	2	3	4	5	6	7	8	9	10	11	12
A	X	X	X	X	X	X	X	X	X	X	X	X
B	X	blank	blank	blank	X	8-500, 200 µM	8-500, 200 µM	8-500, 200 µM	4-300, 200 µM	4-300, 200 µM	4-300, 200 µM	X
C	X	untreated	untreated	untreated	X	8-500, 100 µM	8-500, 100 µM	8-500, 100 µM	4-300, 100 µM	4-300, 100 µM	4-300, 100 µM	X
D	X	stapled P9, 100 µM	stapled P9, 100 µM	stapled P9, 100 µM	X	8-300, 200 µM	8-300, 200 µM	8-300, 200 µM	4-arm, 100 µM	4-arm, 100 µM	4-arm, 100 µM	X
E	X	16-300, 200 µM	16-300, 200 µM	16-300, 200 µM	X	8-300, 100 µM	8-300, 100 µM	8-300, 100 µM	8-arm, 100 µM	8-arm, 100 µM	8-arm, 100 µM	X
F	X	16-300, 100 µM	16-300, 100 µM	16-300, 100 µM	X	4-500, 200 µM	4-500, 200 µM	4-500, 200 µM	unstapled P9, 100 µM	unstapled P9, 100 µM	unstapled P9, 100 µM	X
G	X	short 8-arm, 100 µM	short 8-arm, 100 µM	short 8-arm, 100 µM	X	4-500, 100 µM	4-500, 100 µM	4-500, 100 µM	untreated	untreated	untreated	X
H	X	X	X	X	X	X	X	X	X	X	X	X

Figure S26. Representative picture of the well-plate used for alamarBlue assay. The picture was taken after the fluorescence reading, then overnight incubation at room temperature. X represents water as surrounding wells.

AlamarBlue Assay - polymer control and comb-like conjugates at 50 μM peptide equivalent concentration

In addition to the polymer control, we conducted a further alamarBlue test on conjugates 8-500, 8-300, and 16-300 at a lower peptide equivalent concentration (50 μM). This test showed that 8-300 and 16-300 conjugates completely kill all bacteria, underscoring the high antimicrobial activity of the comb-like conjugates. While the results are promising, we note that the alamarBlue assay at this lower concentration was performed just once (n = 1) with triplicate (3 wells) from 1 solution so we hesitate to draw further conclusions.

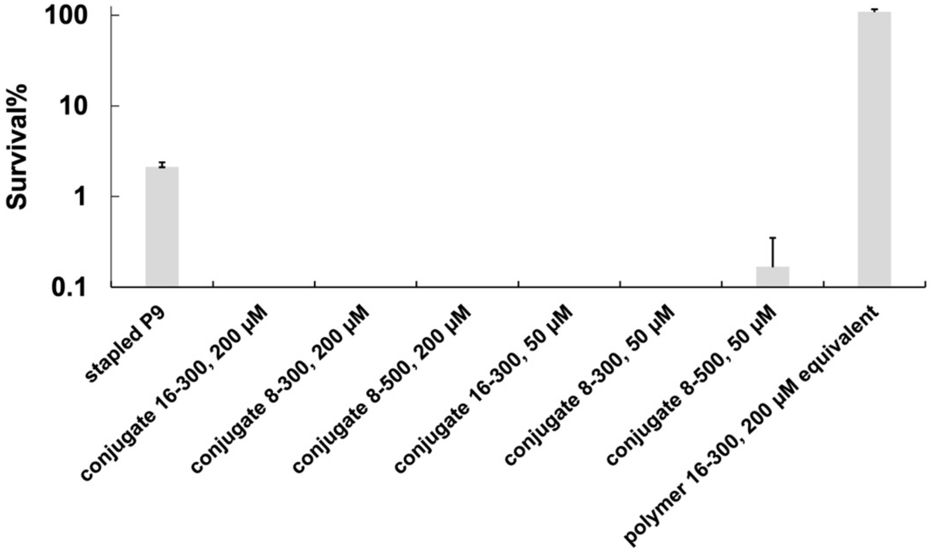

Figure S27. Bacterial survival (%) after treatment with indicated AMP, conjugate (peptide equivalent concentrations = 200 μM or 50 μM in RPMI medium), or polymer. The assays were performed once, with triplicates (3 wells) from 1 polymer solution. Bars indicate the average percent survival. Error bars represent the standard deviation. ND: none detected (bacterial survival < .01%).

Hemolysis

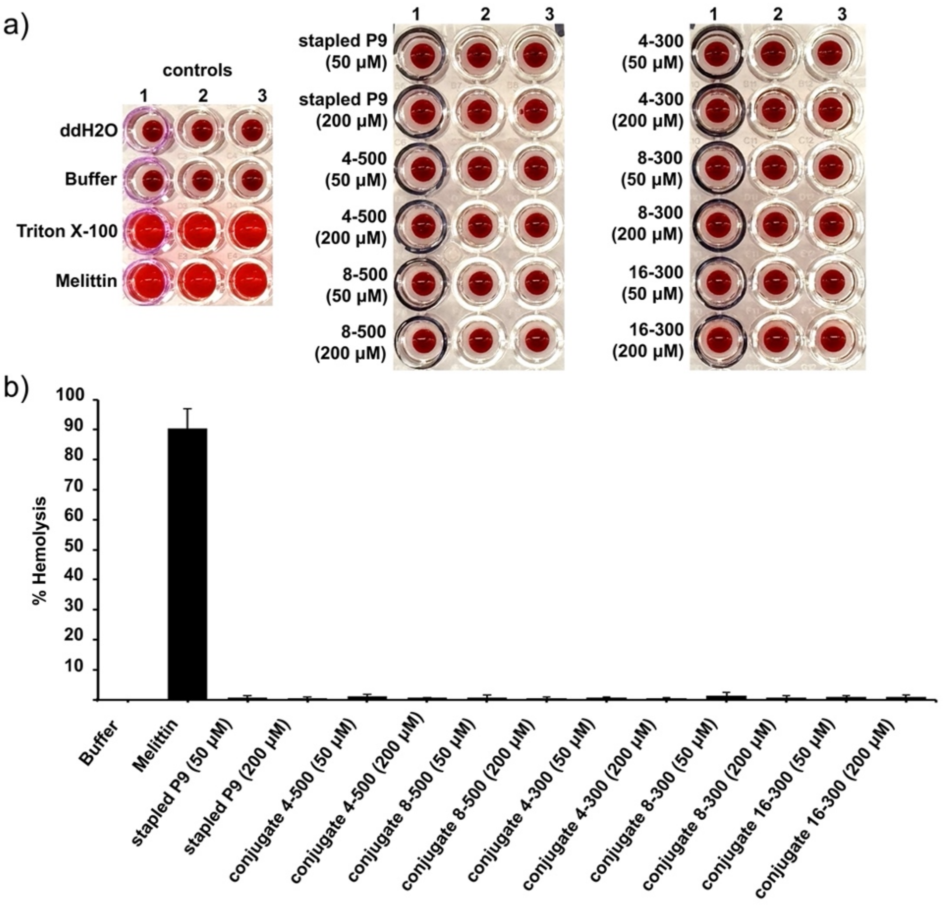

Figure S28. Hemolysis assays of stapled P9 and the corresponding comb-like conjugates. a) representative picture of the well-plate. b) hemolysis percentage. Measurements were performed in triplicate. Error bars represent the standard deviation.
